# The use of human papillomavirus DNA methylation in cervical intraepithelial neoplasia: A systematic review and meta-analysis

**DOI:** 10.1016/j.ebiom.2019.10.053

**Published:** 2019-11-12

**Authors:** Sarah J Bowden, Ilkka Kalliala, Areti A Veroniki, Marc Arbyn, Anita Mitra, Kostas Lathouras, Lisa Mirabello, Marc Chadeau-Hyam, Evangelos Paraskevaidis, James M Flanagan, Maria Kyrgiou

**Affiliations:** a Department of Surgery and Cancer, 3rd Floor IRDB, Faculty of Medicine, Imperial College London, Hammersmith Campus, Du Cane Road, London, W12 ONN, London, UK; b West London Gynaecology Cancer Centre, Hammersmith Hospital, Imperial Healthcare NHS Trust, UK; c Department of Obstetrics and Gynaecology, University of Helsinki and Helsinki University Hospital, Finland; d Li Ka Shing Knowledge Institute, St. Michael's Hospital, Toronto, Canada; e Department of Primary Education, School of Education, University of Ioannina, Ioannina, Greece; f Unit of Cancer Epidemiology, Scientific Institute of Public Health, Brussels, Belgium; g Department of Clinical Genetics, National Institute of Health (NIH), Bethesda, MD, USA; h Department of Obstetrics and Gynaecology, University Hospital of Ioannina, Ioannina, Greece

**Keywords:** Human papillomavirus, DNA methylation, Cervical intraepithelial neoplasia, Cervical screening, Meta-analysis

## Abstract

**Background:**

Methylation of viral DNA has been proposed as a novel biomarker for triage of human papillomavirus (HPV) positive women at screening. This systematic review and meta-analysis aims to assess how methylation levels change with disease severity and to determine diagnostic test accuracy (DTA) in detecting high-grade cervical intra-epithelial neoplasia (CIN).

**Methods:**

We performed searches in MEDLINE, EMBASE and CENTRAL from inception to October 2019. Studies were eligible if they explored HPV methylation levels in HPV positive women. Data were extracted in duplicate and requested from authors where necessary. Random-effects models and a bivariate mixed-effects binary regression model were applied to determine pooled effect estimates.

**Findings:**

44 studies with 8819 high-risk HPV positive women were eligible. The pooled estimates for positive methylation rate in HPV16 L1 gene were higher for high-grade CIN (≥CIN2/high-grade squamous intra-epithelial lesion (HSIL) (95% confidence interval (95%CI:72·7% (47·8–92·2))) vs. low-grade CIN (≤CIN1/low-grade squamous intra-epithelial lesion (LSIL) (44·4% (95%CI:16·0–74·1))). Pooled difference in mean methylation level was significantly higher in ≥CIN2/HSIL vs. ≤CIN1/LSIL for HPV16 L1 (11·3% (95%CI:6·5–16·1)). Pooled odds ratio of HPV16 L1 methylation was 5·5 (95%CI:3·5–8·5) for ≥CIN2/HSIL vs. ≤CIN1/LSIL (*p* < 0·0001). HPV16 L1/L2 genes performed best in predicting CIN2 or worse (pooled sensitivity 77% (95%CI:63–87), specificity 64% (95%CI:55–71), area under the curve (0·73 (95%CI:0·69–0·77)).

**Interpretation:**

Higher HPV methylation is associated with increased disease severity, whilst HPV16 L1/L2 genes demonstrated high diagnostic accuracy to detect high-grade CIN in HPV16 positive women. Direct clinical use is limited by the need for a multi-genotype and standardised assays. Next-generation multiplex HPV sequencing assays are under development and allow potential for rapid, automated and low-cost methylation testing.

**Funding:**

NIHR, Genesis Research Trust, Imperial Healthcare Charity, Wellcome Trust NIHR Imperial BRC, European Union's Horizon 2020

## 1 Introduction

Human papillomavirus (HPV) infects over 70% of sexually active women during their lifetime [1]. In most women, these infections are cleared whilst in a minority these persist and have the potential to lead to invasive cervical cancer [2]. Establishing that

**Research in context**

**Evidence before this study**

The results of studies exploring the use of methylation of viral DNA as a novel proposed biomarker in cervical cancer prevention have been encouraging. Variation in the explored HPV genotypes, genes and adjacent CpG sites across studies together with differences in sequencing techniques and thresholds of positivity make comparisons and interpretations difficult. Although HPV methylation has been proposed as a possible candidate triage marker in HPV positive women at screening, the diagnostic accuracy of the test is not yet established. We aimed to determine how methylation levels change with cervical preinvasive disease grade and its diagnostic accuracy in predicting high-grade cervical precancer in HPV positive women. Electronic bibliographic databases MEDLINE, EMBASE and CENTRAL from 1st January 1990 to 1st October 2019 were searched for published studies, investigating changes in methylation or diagnostic test accuracy, that reported the proportion methylation, mean (or median) methylation level, odds ratio or sensitivity, specificity or AUC. We used search terms including “Uterine Cervical Neoplasm”, “Cervical intraepithelial Neoplasia”, “Uterine Cervical Dysplasia”, “papillomaviradae”, “HPV”, “methylation”, “epigenetics”. There was no language restriction

**Added value of this study**

To date, this is the first comprehensive systematic review and meta-analysis that explores the role of viral methylation in cervical disease. We identified 44 studies and 8819 women and included all HPV genotypes, genes and CpG loci. We found that positive methylation rate and mean methylation increased with increasing disease severity for most genes with the most marked difference in the L1 and L2 region, with no difference in the LCR promoter/enhancer region. The pooled difference in mean methylation level was significantly higher ≥CIN2/HSIL when compared to ≤CIN1/LSIL, and the difference was more marked for the HPV16 L1 gene (11·3%). HPV16 L1/L2 gene performed best in predicting CIN2 or worse in HPV 16 positive women (pooled sensitivity 77%, specificity 64%, AUC 0·73). There were however limitations including substantial residual heterogeneity and the results should be interpreted with caution. The included studies used different test positive criteria, sequencing tests, techniques, and reference verification standards. We performed a series of sensitivity analyses to reduce heterogeneity.

**Implications for all the available evidence**

This study suggests that HPV methylation may be a valuable epigenetic molecular determinant in cervical cancer prevention. The results summarise relative estimates for different HPV methylation sites, genes and combinations and inform the research community of the highest performing genes and sites that should be further explored in future studies. Standardisation of threshold positivity and sequencing protocols is needed to allow valuable comparisons. The diagnostic accuracy of HPV16 L1/L2 in detecting high-grade CIN in HPV16 positive women outperformed most available triage tests, although this was limited by the absence of a multiplex assay that would include all hrHPV types. Such next-generation sequencing assays are being developed and have the potential to allow rapid, automated and low-cost methylation testing. When available, clinical trials should perform head-to-head comparisons with other currently available triage tests in screening populations, to determine those women that warrant referral to colposcopy. The predictive value of viral methylation in infection and/or disease with true carcinogenic potential should also be explored in longitudinal cohorts.

**CRediT authorship contribution statement**

**Sarah J Bowden:** Conceptualization, Investigation, Data curation, Writing - review & editing, Formal analysis. **Ilkka Kalliala:** Investigation, Methodology, Writing - review & editing, Formal analysis. **Areti A Veroniki:** Writing - review & editing, Methodology, Formal analysis. **Marc Arbyn:** Software, Writing - review & editing. **Anita Mitra:** Data curation, Writing - review & editing. **Kostas Lathouras:** Data curation, Writing - review & editing. **Lisa Mirabello:** Writing - review & editing. **Marc Chadeau-Hyam:** Writing - review & editing. **Evangelos Paraskevaidis:** Writing - review & editing. **James M Flanagan:** Writing - review & editing. **Maria Kyrgiou:** Conceptualization, Methodology, Supervision, Writing - review & editing.

Alt-text: Unlabelled Box

persistent infection with high-risk oncogenic HPV is causally associated with cervical cancer has led to major advances in cervical cancer prevention.

High-risk HPV (hrHPV) testing for primary cervical screening may offer 60–70% greater protection against invasive cancer than cytology-based screening [3]. The estimated HPV prevalence in screened populations varies from 9–15%, often reaching more than 30% in women aged less than 30 [4], and in most cases infection is transient. HPV testing has modest specificity and positive predictive value (PPV) for the detection of high-grade cervical intra-epithelial neoplasia (CIN) [5]. The best strategy to triage HPV positive women at screening and detect those truly at risk remains unclear.

Novel molecular tests based on the HPV genome and epigenome have the potential to permit a more comprehensive understanding of the disease process and as a result improve diagnostic accuracy within cervical screening programmes. DNA hyper-methylation is an early and frequent event in many cancers and methylation of viral DNA has been recently proposed as a novel biomarker of cervical disease. Rapid developments in sequencing technologies have enhanced scientific ability to explore the HPV epigenome, together with its host interactions. Consequently, the number of studies investigating the role of HPV DNA methylation in disease progression has increased in recent years, the most comprehensive of which suggest an increase in methylation by disease grade in common high-risk HPV types [6], [7], [8], [9].

Although results are encouraging [10], variation in measurement and reporting of methylation has made comparisons difficult. Studies examining genome-wide HPV methylation have thus far been limited and methylation levels appear to vary by HPV genotype, gene (L1, L2, E2-4, URR) and even amongst adjacent CpG sites [11,12]. Differences in case definitions and endpoints (e.g. cytology vs. histopathology, CIN2 vs. CIN3) further complicate comparisons and interpretation of the literature. To date, current evidence has not permitted analysis of the comparative performance of different HPV methylation sites. To this end, we conducted a systematic review and meta-analysis of the existing literature with the aim to explore how methylation levels of different HPV genotypes and genes correlate with disease severity (CIN histological grade) and to further determine the diagnostic accuracy of HPV methylation in detecting high-grade pre-invasive disease in HPV positive women.

## 2 Materials and methods

### 2.1 Selection criteria and search strategy

The meta-analyses followed Preferred Reporting Items for Systematic Reviews and Meta-Analyses (PRISMA) guidelines and Cochrane Collaboration recommendations for diagnostic test accuracy reviews [13,14]. Ethical approval was not required. A systematic literature search was performed by an experienced Cochrane librarian in the electronic bibliographic databases MEDLINE, EMBASE and CENTRAL from inception to 1st October 2019. The full search strategy is presented in the supplementary methods. Studies were identified by two independent reviewers (SB, AM) and discrepancies resolved by a third author (IK). To identify studies which might have been missed during the electronic search and unpublished data, we further hand-searched the references of the articles in the full-text stage and the proceedings of relevant conferences respectively.

We included all studies examining the degree of HPV DNA methylation across the viral epigenome with HPV infection and different grades of cervical disease as defined by cytology or histopathology. Any population of women representing a cervical screening or referral population were included without demographic restrictions, including retrospective biobanks of samples for women recruited from such populations and diagnostic accuracy studies examining the ability of HPV methylation to diagnose cytological or histological outcomes, regardless of HPV genotype, sample material and study methodology. We excluded studies where a reference standard (cytological and/or histological grade) was not reported and methylation experiment studies in cell lines or tumor clones only, due to low applicability to cervical screening populations. Studies with less than five participants with CIN2 or worse were excluded. There was no language restriction.

### 2.2 Outcome measures, data extraction and risk of bias

We explored two primary outcomes. Firstly, we established how gene-specific HPV methylation changes with CIN severity. The reported outcome measures presented across studies varied and included (a) proportion of samples methylated at a CpG and/or gene (samples methylated/samples methylated plus samples unmethylated) (b) percentage mean or median methylation at a CpG and/or gene (0–100% methylation) (c) mean difference (MD) in methylation (mean methylation in high-grade disease minus mean methylation in low-grade disease) d) odds-ratio (OR) of high-grade vs. low-grade disease for high methylation (supplementary methods). Secondly, we determined the diagnostic accuracy of a positive HPV methylation test (sensitivity, specificity or area under the curve (AUC)) in detecting ≥CIN2 and ≥CIN3.

We assessed the risk of bias for selected studies using a modified quality assessment tool for diagnostic test accuracy studies (QUADAS-2) [15,16] which covers four main domains: patient selection, index test, reference standard, flow and timing (supplementary methods). We assigned a judgement of low, moderate or high risk of bias to each of the domains according to the criteria outlined in QUADAS-2 and the Cochrane Handbook [13].

From each included study, two reviewers (SB, AM) extracted data on study design and setting, index test, sample material, HPV type, CpG sites analysed, reference standard, outcome measures and risk of bias using a predefined spreadsheet (supplementary methods).

### 2.3 Statistical analysis

We analysed methylation changes with disease severity for the different outcome measures reported across studies; pooled estimates of study-specific means, proportions, MD and OR were calculated using an inverse variance random-effects meta-analysis model in the metafor R package v3.6.1. Our main analysis compared two groups based on standardised classification systems. CIN and squamous intraepithelial lesion (SIL) classifications have been commonly used to describe cervical disease (cytology and histology) and the choice has interchanged between different time periods and continents. Similar grades of disease from either terminology were therefore grouped together: women with ≥CIN2/HSIL (histologically confirmed CIN2 or worse/cytologically defined high-grade squamous intra-epithelial lesion (HSIL) or worse) vs. ≤CIN1/LSIL (histologically confirmed CIN1 or less/ cytologically defined low-grade squamous intra-epithelial lesion (LSIL) or less). We used histopathology to determine CIN grade if available; if histopathology (CIN) was not available or clinically indicated, the cytology result (SIL) was used. Terminology is further described in the supplementary glossary.

We compared the proportions of methylated samples for ≥CIN2/HSIL and ≤CIN1/LSIL in a meta-analysis using the variance-stabilising Freeman–Tukey double arcsine transformation approach. We used the individual author definition of methylated samples. We performed secondary analyses in more defined clinical groups to include normal, LSIL, HSIL and invasive cervical cancer (ICC).

From extracted mean methylation levels we calculated the study-specific MD. ORs were extracted from studies along with their variances, but when these were not available, they were estimated from proportions methylated or transformed from continuous data. We performed assessment for publication bias in the main analysis by visual assessment of the funnel plot and Egger's *P*-value for small study effects [17].

Pooled absolute sensitivity and specificity were estimated jointly in a bivariate model using the midas routine in Stata v.14, taking the correlation between true-positive and false-positive rates and between-study variability into account. Where insufficient data were available, we contacted authors for data by individual CpG site and varying cut-offs. Pre- and post-test probability analyses were calculated using the ppp function [18]. We used the diagnostic accuracy of the best performing HPV type, gene and CpG sites. Pre-test probability of CIN2/HSIL or worse in women with HPV16 was estimated from recently presented data [19].

We assessed between-study heterogeneity using the Cochran's Q test, a visual inspection of forest plots, and the *I^2^* statistic along a 95% confidence interval (CI) [20]. We estimated the between-study variance using the restricted maximum likelihood approach in R and the DerSimonian and Laird method in Stata.

We performed predefined sensitivity analyses. For the comparison of proportions and mean methylation by disease grade, we restricted to those that used histopathology alone as a reference (cytology was accepted to define normal alone). For the MD, OR and diagnostic accuracy analyses, we restricted to (a) studies at low risk of bias; (b) histopathology as reference test (c) pyrosequencing as index test (d) cervical exfoliated cells (CEC) as sample material (e) cut-off limited to 10% mean methylation. In the MD meta-analysis, we additionally excluded imputed SD values for sensitivity analysis, whereas in the OR meta-analysis, we also excluded the transformed OR values from continuous data and performed a sub-group analysis by geographical region.

More details on the methods are described in the supplement and in our protocol, which was registered with Open Science Framework.

## 3 Results

The literature search yielded 572 potentially eligible studies; 44 studies [6], [7], [8], [9],[21], [22], [23], [24], [25], [26], [27], [28], [29], [30], [31], [32], [33], [34], [35], [36], [37], [38], [39], [40], [41], [42], [43], [44], [45], [46], [47], [48], [49], [50], [51], [52], [53], [54], [55], [56], [57], [58], [59], [60] and 8819 women were included in the analysis (supplementary table 1, supplementary figure 1).

The methodological quality of the studies was overall moderate (Table 1). Half were at low-risk of bias, (50·0%, 22/44), whilst the rest had either moderate (45·5%, 20/44) or high risk (4·7%, 2/44). For patient selection, 43·2% (19/44) studies scored moderate to high risk of bias as samples were often selected from biobanks, case-controls without consecutive recruitment, with some having inappropriate exclusions. The index test scored moderate or high risk of bias in 34·1% (15/44); reasons included use of older bisulphite sequencing methods, no quantitative measure, and use of vaginal samples. Eighty-six percent (38/44) of studies scored high or moderate risk of bias on the reference test as there was no blinding of the person performing methylation, while in 13·6% (6/44) there was no histological confirmation for high-grade disease. For flow and timing, there was a delay between methylation and reference confirmation in 9·0% (4/44) of studies, whilst differential verification bias was present in 50·0% (22/44), as studies switched between cytology and histopathology to confirm disease grade. 38·6% (17/44) of studies explained withdrawals or uninterpretable results adequately, while 61·3% (27/44) were scored unclear for insufficient explanation.

**Table 1:** Quality assessment for risk of bias for all included studies with tailored QUADAS-2.

*QUADAS-2 domains.* P1 = acceptable enrolment method; P2 = acceptable inclusion criteria and inappropriate exclusions avoided; I1 = acceptable sample material (LBC, cervical swab, cervical tissue); I2 = Acceptable methylation test giving quantitative results (Pyrosequencing, EpiTYPER, Next generation sequencing, Luminex-C); R1 = Acceptable reference test (histology confirmation of grade for CIN and cancer, at least cytology confirmation of grade for normal and ascus); R2 = Masking of methylation analysis to reference test; F1 = Acceptable interval between index test and reference standard?; F2 = Differential verification avoided; F3 = withdrawals and uninterpretable results explained. Subdomains were assessed according to QUADAS-2 guidance by answering yes (green tick), no (red cross) or unclear (yellow exclamation mark), where unclear relates to insufficient data for assessment). Domains were assessed by summarising results of subdomains, into high, moderate or low risk of bias.

All studies were observational. Forty reported on HPV16, twelve on HPV18, and twelve on other HPV types. The majority of studies explored the L1 gene (*N* = 30), followed by LCR (*N* = 29), L2 (*N* = 15), E6 (*N* = 10), E2 (*N* = 4), E7 (*N* = 3), E5 (*N* = 2) and E1 (*N* = 2). Recent studies used pyrosequencing (27/44, 61·3%) and next generation sequencing (NGS) techniques (4/44, 9·0%) for further quantification. Cut-offs of positivity varied ranging from 0 to 90%, whilst some studies chose dichotomisation after division by tertiles. Seventeen studies (39·5%) reported histopathological diagnosis for all samples, twenty-two (50·0%) histopathology for ≥CIN2/HSIL and cytology alone for ≤CIN1/LSIL, three studies (7·0%) used cytology for CIN but histopathology for cancer [26,^31^,32], one study used cytology alone [49], and two studies did not define the reference test [33,40]. The majority used exfoliated cervical cells (40/44, 90·9%). Assessment for publication bias revealed some asymmetry with large studies on the left showing a more conservative estimate, while Egger's P of <0·01 indicated small study effects (supplementary figure 2).

### 3.1 Methylation and lesion severity

Within studies reporting proportions of methylated samples according to disease grade, we found that rates of positive methylation increased with disease grade. When we compared women with ≤CIN1/LSIL to ≥CIN2/HSIL the most marked difference in the pooled estimates for positive methylation rate were found at the HPV16 L1 gene (8 studies; pooled positive methylation rate: ≤CIN1/LSIL = 44·4% (95%CI 16·0–74·1) and ≥CIN2/HSIL = 72·7% (95%CI 47·8–92·2) (Table 2).

**Table 2:** Meta-analysis of proportion of HPV methylated samples for different genotypes and genes for ≤CIN1/LSIL vs. ≥CIN2/HSIL. ≤CIN1/LSIL: Normal/ASCUS/LSIL/CIN1. ≥CIN2/HSIL: CIN2/CIN3/HSIL/ICC. The grade was defined by histology; if this was not available, cytology was used.

Author, year	Cut-off	CpG	Study size	≤CIN1/LSIL% (N/N)	≥CIN2/HSIL% (N/N)
**HPV16 L1**				**%**	**%**
Brentnall 2014	>0%	L1 (bp6367, 6389)	556	90.1 (210/233)	97.8 (326/323)
Qiu 2015 (1)	>10%	L1 (bp5602, 5608, 5611, 5617)	114	40.5 (17/42)	91.6 (66/72)
Qiu 2015 (2)	>10%	L1 (bp7136, 7145)	81	34.3 (11/32)	89.8 (44/49)
Simanaviciene 2015	>0%	L1 (bp7136, 7145)	157	2.6 (1/39)	24.6 (29/118)
Gasperov 2015	>0%	L1 (bp7091, 7136, 7145, 7145)	16	30 (3/10)	33.3 (2/6)
Kalantari 2014	>0%	L1/L2 (bp 5602–7270)	63	13.8 (4/29)	50 (17/34)
Lorincz 2013	>0%	L1 (bp 6367,6389)	73	100 (48/48)	100 (25/25)
Wang 2017	>0%	L1 (bp 7089–7268)	100	16.6 (5/30)	50 (35/70)
Total *N* – *n*/N*			**1160**	**299/463**	**534/697**
**Pooled estimate (95% CI)**				**44.4 (16.0–74.1)**	**72.7 (47.8–92.2)**
*I^2^* (95%CI)				97.61 (93.2–99.3)	97.2 (93.3–99.3)
tau^2^ (95%CI)				0.18 (0.07–0.75)	0.11 (0.04–0.41)
					
**HPV 16 L2**					
Brentnall 2014	>0%	L2 (bp4238, 4259, 4275)	556	29.3 (68/233)	55 (178/323)
Kalantari 2014	>0%	L1/L2 (bp 5602–7270)	63	13.8 (4/29)	50 (17/34)
Lorincz 2013	>0%	L2 (bp 4238, 4247, 4259, 4268, 4275)	73	79 (38/48)	96 (24/25)
**Total *N* – *n*/*N***			**692**	**110/310**	**219/382**
**Pooled Estimate (95% CI)**				**40.1 (7–79.3)**	**61.8 (43.9–78.2)**
*I^2^* (95%CI)				96.9 (88.1–99.9)	85.2 (39.6–99.7)
tau^2^ (95%CI)				0.12 (0.03–5.12)	0.02 (0.00–1.04)
					
**HPV16 LCR**					
Badal 2003	>0%	5′ LCR, Enh, E6 Prom	81	42.1 (16/38)	9.3 (4/43)
Bhattarcharjee 2006	>0%	5′LCR (bp7289-7540)	72	4 (6/15)	54.4 (31/57)
Ding 2009	>0%	5′LCR, Enh, E6 Prom (bp7426-58)	53	5.9 (1/17)	41.7 (15/36)
Dutta 2015	>0%	Enh (bp7535-7694), E6 Prom	215	4.1 (41/93)	44.3 (54/122)
Hublarova 2009	>0%	E6 Prom (bp7851-7559)	141	81 (17/21)	35.8 (43/120)
Gasperov 2015	>0%	3′LCR, Enh, Prom (bp7091-58)	12	50 (3/6)	33.3 (2/6)
Simanaviciene 2015	>0%	5′LCR, Enh, Prom (bp7270-58)	157	2.7 (1/39)	23.7 (28/118)
Xi 2011	>0%	3′LCR, Enh, Prom (bp7535-58)	211	44.9 (53/117)	35.6 (34/94)
Snellenberg 2012	>0%	E2BS1 (bp 7370–7383)	65	11.8 (2/17)	64.6 (31/48)
Hong 2008	>0%	Enh, Prom (bp7676-58)	70	48.4 (15/31)	71.8 (28/39)
Lorincz 2013	>0%	Prom (bp31-58)	73	90 (43/48)	92 (23/25)
Wang 2017	>0%	3′L1, 5′LCR, Enh, Prom (bp7089-58)	101	8.8 (3/34)	32.8 (22/67)
**Total *N* – *n*/*N***			**1251**	**201/476**	**315/775**
**Pooled Estimate (95% CI)**				**37.5 (20.2–56.5)**	**44.5 (30.9–58.5)**
*I^2^* (95%CI)				93.4 (86.4–97.7)	92.8 (85.1–97.6)
tau^2^ (95%CI)				0.09 (0.05–1.04)	0.05 (0.02–0.16)
					
**HPV 18 L1/L2**					
Simanaviciene 2015	>0%	L1 (bp6916-7122)	21	18.2 (2/11)	80 (8/10)
Gasperov 2015	>0%	L1 (bp7017-7140)	21	100 (9/9)	92.3 (12/13)
Brentnall 2014	>0%	L2 (bp4256-4282)	201	70.9 (91/128)	84.1 (61/73)
Kalantari 2014	>0%	L1/L2 (bp5602-7270)	14	14.3 (1/7)	85.7 (6/7)
**Total *N* – *n*/*N***			**258**	**103/155**	**87/103**
**Pooled Estimate (95% CI)**				**52.6 (13.8–89.8)**	**86.0 (77.7–92.8)**
*I^2^* (95%CI)				90.6 (67.7–99.4)	0.0 (0.0–75.4)
tau^2^ (95%CI)				0.14 (0.03–2.30)	0.00 (0.00–0.05)
					
**HPV18 LCR**					
Simanaviciene 2015	>0%	LCR (LCR5′, Prom, Enh)	21	0 (0/13)	20 (2/8)
**Total *N* – *n*/*N***			**21**	**0/13**	**2/8**
					
**HPV 31 L1/L2**					
Brentnall 2014	>0%	L1 (bp6352, 6364)	202	59.6 (62/104)	85.7 (84/98)
Kalantari 2014	>0%	L1/L2 (bp5518-5692)	15	0 (0/7)	50 (4/8)
**Total *N* – *n*/*N***			**217**	**62/111**	**88/106**
**Pooled Estimate (95% CI)**				**33.6 (0.0–87.1)**	**73.7 (34.4–99.3)**
*I^2^* (95%CI)				89.6 (47.6–100)	89.6 (47.6–100)
tau^2^				0.14 (0.01–100)	0.06 (0–78.5)
					
**HPV 45 L1/L2**					
Kalantari 2014	>0%	L1/L2 (bp4795-7135)	12	0 (0/1)	80 (8/10)
**Total *N* – *n*/*N***			**12**	**0/1**	**8/10**
					
**HPV 52 L1**					
Murakami 2013	>0%	L1 (bp5606-7120)	54	15 (2.6/17)	44.7 (17/37)
**Total *N* – *n*/*N***			**54**	**2.6/17**	**17/37**
					
**HPV 52 LCR**					
Murakami 2013	>0%	LCR (LCR5′, Prom, Enh)	54	2.5 (0.4/17)	2.4 (0.9/37)
**Total *N* – *n*/*N***			**54**	**0.4/17**	**0.9/37**
**HPV 58 L1**					
Murakami 2013	>0%	L1 (bp5606-7119)	41	12 (2.4/20)	51.1 (10.7/21)
**Total *N* – *n*/*N***			**41**	**2.4/20**	**10.7/21**
					
**HPV 58 LCR**					
Murakami 2013	>0%	LCR (LCR5′, Prom, Enh)	41	0 (0/20)	0 (0/21)
**Total *N* – *n*/*N***			**41**	**0/20**	**0/21**

ASCUS: abnormal squamous cells of undetermined significance: Bp: base pair; CIN: cervical intraepithelial neoplasia; Enh: enhancer region; HSIL: high-grade squamous intraepithelial lesion; LCR: long control region; LSIL: low-grade squamous intraepithelial lesion; *N*: total number of samples; *N*: number of samples methylated; ICC: invasive cervical cancer Prom: promoter region; Q-test: Cochrane Q test.

Proportion of samples methylated at a CpG and/or gene was defined as: *N* methylated/*N* methylated + *N* unmethylated.

Where studies presented results for multiple CpG sites per gene, the mean result for the gene was meta-analysed.

The contrast was consistent for HPV16 L2 gene, although the number of studies was smaller and between study heterogeneity was high (3 studies; 40·1% (95%CI 7·0–79·3) vs. 61·8% (95%CI 43·9–78·2)), whereas the differences between ≤CIN1/LSIL vs. ≥CIN2/HSIL were much smaller at the HPV16 LCR (12 studies; 37·5% (95%CI 20·2–56·5); vs. 44·5% (95%CI 30·9–58·5)). The differences were also marked for HPV 18 L1/L2 genes (4 studies; 52·6% (95%CI 13·8–89·8) vs. 86·0% (95%CI 77·7–92·8)) and for HPV31 L1/L2 (2 studies, 33·6% (95%CI 0·0–87·1) vs. 73·7% (95%CI 34·4–99·3)). There were not sufficient studies for pooled estimates for other HPV types (45,52,58), although the direction of effect was similar in the individual studies. We further analysed the data in more defined groups of normal, CIN1/LSIL, CIN2-3/HSIL, and invasive cervical cancer (ICC) and similar associations were observed (supplementary table 2). The sensitivity analysis using studies that had histopathology alone as a reference standard were analysed separately (supplementary table 3). We found consistent results with reduced heterogeneity and a more marked difference in the methylated rates for HPV16 L1 in ≥CIN2 vs. ≤CIN1 (71·5% (95%CI 24·8–99·8) vs. 24·1% (95%CI 5·3–50·0), suggesting that cytology as a reference may have misclassified disease. The quantification of the difference in mean methylation levels showed that HPV16 methylation was significantly higher in ≥CIN2/HSIL vs. ≤CIN1/LSIL in individual studies in all genes (*P* < 0·05) except the LCR (*P* = 0·169) (supplementary figure 3a). The pooled mean methylation levels for HPV16 were lower overall in women with CIN1/LSIL or less when compared to those with CIN2/HSIL or more (Table 3).

**Table 3:** Pooled mean methylation levels for HPV16 in ≤CIN1/LSIL vs. ≥CIN2/HSIL and pooled difference in mean methylation (MD). The grade was defined by histology; if this was not available, cytology was used.

Gene	Studies (*N*)	Pooled mean % (95%CI)	*I^2^* (95%CI)	tau^2^ (95%CI)	Pooled mean % (95%CI)	*I^2^* (95%CI)	tau^2^ (95%CI)	Pooled MD % (95%CI)	*I^2^* (95%CI)	tau^2^ (95%CI)
		≤CIN1/LSIL			≥CIN2/HSIL					

E1	1	3.9 (2.1–5.7)	N/A	N/A	14.8 (9.1–20.4)	N/A	N/A	4.0 (−1.6–9.6)	N/A	N/A
E2	4	5.6 (1.6–9.5)	89.0 (60.2–98.8)	13.7 (2.6–137)	14.1 (5.4–22.8)	87.9 (62.6–98.6)	67.9 (15.7- 679)	8.8 (0.2–17.5)	88.0 (61.5–98.7)	67.5 (14.7–675)
E5	1	14.0 (9.8–18.1)	N/A	N/A	26.6 (17.7–35.5)	N/A	N/A	12.7 (2.9–22.4)	N/A	N/A
E6	3	2.1 (0.8–3.4)	72.0 (5.1–99.2)	0.9 (0.0–42.7)	4.8 (1.7–7.9)	57.0 (0.0–96.9)	4.3 (0–100)	2.5 (0.5–4.4)	13.3 (0.0–96.6)	0.53 (0.00–100)
E7	1	5.9 (4.0–7.9)	N/A	N/A	13.5 (9.1–17.8)	N/A	N/A	7.5 (2.4–2.7)	N/A	N/A
L1	12	10.8 (7.4–14.2)	96.3 (92.2–98.8)	32.9 (14.9–105)	23.3 (17.8–28.7)	96.7 (93.1–98.8)	87.3 (41.1–248)	11.3 (6.5–16.1)	93.0 (85.0–97.6)	63.4 (27.2–196)
L2	5	9.0 (4.8–13.2)	99.4 (98.2–99.9)	22.1 (7.5–186)	15.4 (9.3–21.4)	96.0 (87.8–99.6)	6.6 (3.6–20.3)	5.6 (1.8–9.4)	88.0 (54.3–98.7)	14.5 (2.4–145)
LCR	7	4.0 (1.4–6.7)	96.7 (91.5–99.3)	12.1 (4.5–57.3)	6.6 (4.0–9.3)	94.1 (85.2–98.8)	11.5 (4.1–59.2)	2.5 (−0.1–4.8)	87.3 (64.4–97.9)	8.11 (2.13–54.1)

CIN: cervical intraepithelial neoplasia; LCR: long control region; LSIL: low grade squamous intraepithelial lesion; HSIL: high grade squamous intraepithelial lesion; Q-test: Cochrane Q test. Mean methylation levels in studies were determined by percentage mean or transformed median methylation per group of samples, and averaged by gene (0–100% methylation). Mean difference values in studies were calculated by subtracting mean methylation values from low-grade disease samples from high-grade disease samples, then average by gene (0–100%).

There were insufficient data for pooled analysis of other HPV types but the mean methylation levels for HPV18 L1, HPV18 L2, HPV18 LCR, HPV35 L2, HPV45 E2, HPV45 L1, HPV45 L2 and HPV59 L1 were systematically significantly lower in women with ≤CIN1/LSIL when compared to those with ≥CIN2/HSIL (*P* < 0·05) (supplementary figure 3b). In HPV31 L2, HPV33 L1, HPV33 L2, HPV35 L1, HPV51 L1, HPV51 L2, HPV52 L1, HPV52 L2, HPV58 L1 and HPV58 L2, mean methylation levels in individual studies were higher in ≥CIN2/HSIL but did not reach statistical significance. When restricting to studies using histopathology only as reference, pooled mean methylation levels also increased by disease grade (CIN1, CIN2, CIN3, ICC) in all HPV16 genomic regions (supplementary table 4).

The pooled difference in mean methylation level (MD) was significantly higher in ≥CIN2/HSIL when compared to ≤CIN1/LSIL for the HPV16 L1 gene (11·3% (95%CI 6·5–16·1)) followed by E2 (8·8% (95%CI 0·2–17·5)), E7 (7·7% (95%CI 3·7–11·7)), L2 (5·6% (95%CI 1·8–9·4)) and E6 (2·5% (95%CI 0·5–4·4)) (Table 2, supplementary figure 4). In the LCR there was no significant difference observed (1·6% (95%CI −0·3–3·4)), while for HPV16 E1 and E5 there was only one study with the same direction of effect. High heterogeneity was observed in all MD meta-analyses and was explored with a series of sensitivity analyses that did not substantially improve heterogeneity (supplementary table 5). There were insufficient data for meta-analysis in other HPV genotypes.

**Fig. 1:**
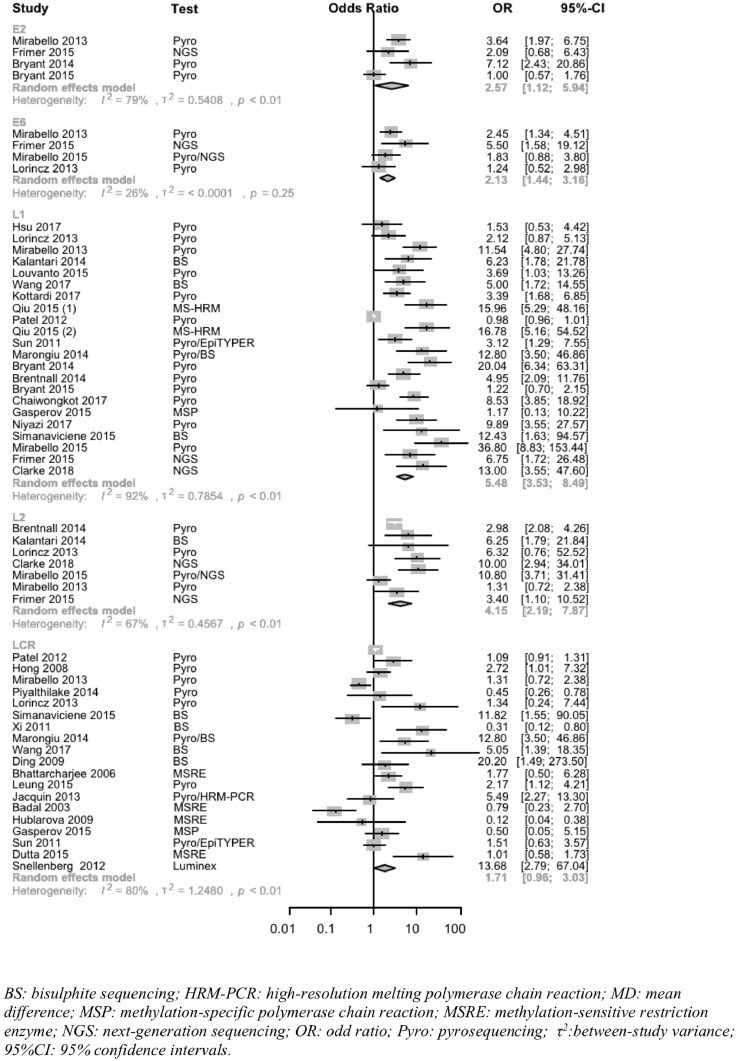
Meta-analysis of odds ratios of HPV16 methylation in genes L1, L2, LCR, E2, E6 for ≤CIN1/LSIL vs. ≥CIN2/HSIL.

Data to determine odds ratio (OR) comparing proportion of methylated samples or mean methylation for ≥CIN2/HSIL vs. ≤CIN1/LSIL in HPV16 was available in 21 studies; the study-specific ORs for HPV16 L1 ranged from 1·2 to 36·8. The cut-off used to define positive methylation varied between studies (range 0–16%). The pooled point estimate OR was greatest for the HPV16 L1 gene (22 studies, OR 5·5 (95%CI 3·5–8·5, *P* < 0·0001)) followed by L2 (7 studies, OR 4·2 (95%CI 2·2–7·8, *P* = 0·0001)), E2 (4 studies, OR 2·6 (95%CI 1·1–6·0, *P* = 0·0266)) and E6 (4 studies, OR 2·1 (95%CI 1·4–3·2, *P* = 0·0002)) (Fig. 1, supplementary Table 7). The OR was not significantly different from unity for the HPV16 LCR region (*P* = 0·0682), with a few studies showing significant negative associations. When restricting the analysis to studies reporting only on proportions, the OR for HPV16 L1 gene was even higher (OR 6·5 (95%CI 3·8–10·9, *P* < 0·0001). Restriction to studies with low risk of bias, histopathology only, or CEC only, all reduced observed heterogeneity. Restriction to pyrosequencing techniques only did not. The pooled OR for HPV18 L1/L2 was similar (7 studies, OR 8·3 (95%CI 2·9–23·5, *P* < 0·001)). Fewer studies were available for other HPV types for the L1 gene alone. Subgroup analysis by geographical location was applied and revealed no significant difference in pooled estimates of OR according continent, including Americas, East Asia, Europe and South Asia (supplementary figure 5).

### 3.2 Diagnostic accuracy in detecting high-grade CIN in women positive for individual high-risk HPV subtypes

**Fig. 2:**
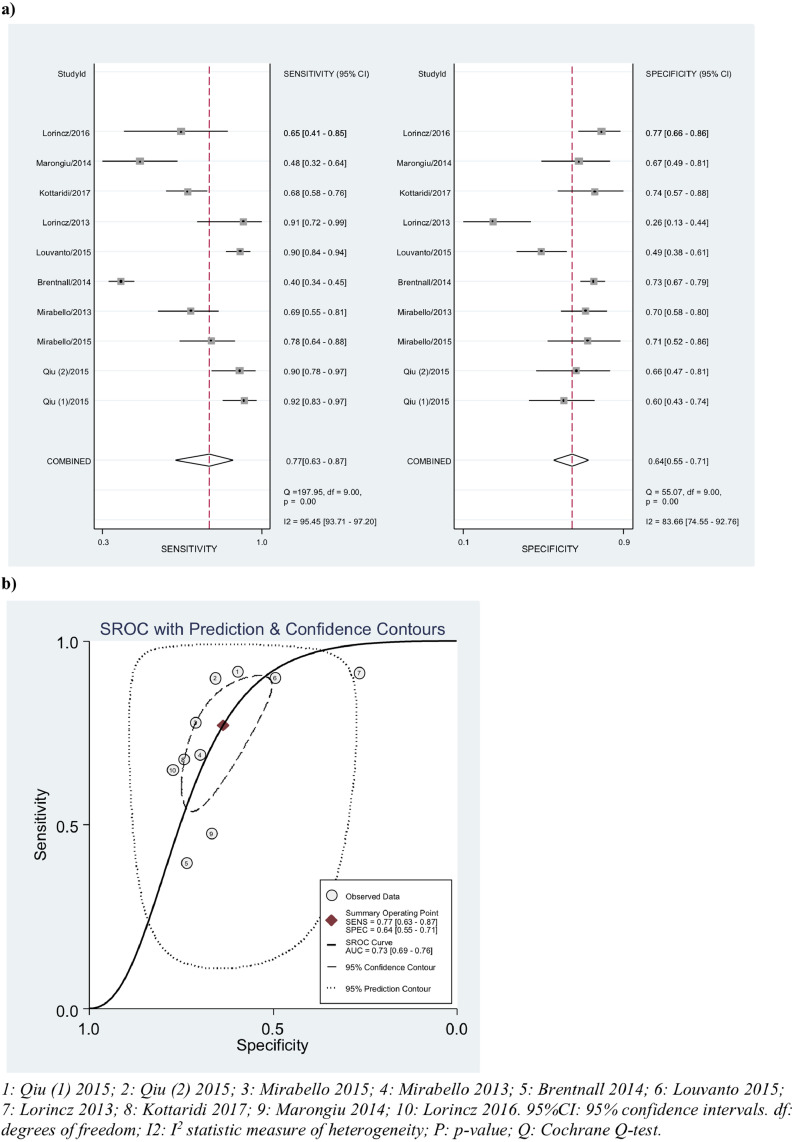
Diagnostic accuracy of HPV16 L1/L2 in predicting CIN2/HSIL or worse (bivariate model) (a) Pooled sensitivity and specificity (b) SROC curve.

We calculated sensitivity and specificity of positive methylation (as defined by authors) in detecting CIN2 or worse and CIN3 or worse by pooling estimates by gene and by best performing CpG site. The cut-off for positive methylation varied across studies (0–15·9% or 3rd tertile). The highest sensitivity and specificity in CIN2+ detection in HPV16 positive women was observed by combination of positive methylation across CpG sites in the HPV16 L1/L2 genes (10 studies, sensitivity 77% (95%CI 63–87), specificity 64% (95%CI 55–71), AUC = 0·73 (95%CI 69–76), *I*^2^ = 96%) (Table 4 and Fig. 2). When all HPV genes were included (L1/L2/E2/E5/E6/E7/LCR) the results were similar (sensitivity 75% (95%CI 59–86), specificity 65% (95%CI 57–73). The pooled estimates for the L1 gene alone had improved sensitivity (82% (95%CI 73–88)) with a slight drop in specificity (57% (95%CI 49–65)). The analysis of L2 alone was similar to that of L1/L2 combined (5 studies). There were sufficient data to analyse pooled sensitivity and specificity of two single CpG site markers (L16367 and L16389) – performance was less than combined gene markers (Table 4). For prediction of CIN3 or worse, sensitivity was similar (76% (95%CI 66–84)), while specificity (75% (95%CI 57–87)) and AUC (0·90 (95%CI 0·80–0·96)) were greater (supplementary figure 9).

**Table 4:** Meta-analysis of HPV16 methylation pooled sensitivity, specificity and area under the curve (by study defined cut-off) in detecting disease **≥**CIN2/HSIL and disease **≥**CIN3/HSIL.

Gene	Studies (*N*)^a^	Threshold (%)	Pooled sensitivity % (95% CI)	Pooled specificity % (95% CI)	Pooled estimated AUC (95% CI)	*I^2^* (95% CI)	tau^2^ (95% CI)
**BIVARIATE MODEL**							
**≥CIN2/HSIL**							
All studies (L1/L2/E2//E5/E6/E7/LCR)	11	0.4–15.9/3rd tertile	75 (59–86)	65 (57–73)	0.73 (0.69–0.76)	95.1 (93–97)	0.76 (0.00–1.89)
L1/L2/E5	11	0.4–15.9/3rd tertile	76 (63–86)	64 (56–72)	0.73 (0.69–0.77)	95.0 (93–97)	0.43 (0.00–1.10)
L1/L2	10	0.4–15.9/3rd tertile	77 (63–87)	64 (55–71)	0.73 (0.69–0.76)	95.5 (94–97)	0.00 (0.00–1.34)
L1	10	0.4–15.9/3rd tertile	82 (73–88)	57 (49–65)	0.73 (0.69–0.77)	85.9 (78–93)	0.00 (0.00–2.99)
L2	5	0.4–15.9/3rd tertile	75 (63–84)	66 (42–84)	0.77 (0.73–0.81)	32.9 (0–98)	0.00 (0.00–100)
L1 6367	5	3.1–10/3rd tertile	74 (64–82)	53 (41–66)	0.70 (0.66–0.74)	73.0 (48–98)	0.35(0.00–0.98)
L1 6389	5	3.1–10/3rd tertile	77 (67–84)	52 (38–65)	0.71 (0.67–0.75)	67.9 (38–98)	0.00 (0.00–1.64)
							
**UNIVARIATE MODEL**							
**≥CIN2/HSIL**							
All studies (L1/L2/E2//E5/E6/E7/LCR)	14	0.4–15.9/3rd tertile	74 (60–84)	65 (54–75)	0.782 (0.669–0.864)	39.5 (0–67)	0.50 (0.00–1.58)
L1/L2/E5	14	0.4–15.9/3rd tertile	75 (62–84)	64 (54–74)	0.810 (0.712–0.880)	37.4 (0–61.7)	0.46(0.00–1.24)
L1/L2	13	0.4–15.9/3rd tertile	75 (61–85)	64 (53–74)	0.808 (0.685–0.891)	39.7 (0.00–67.14)	0.49 (0.00–1.49)
L1	13	0.4–15.9/3rd tertile	81 (73–87)	55 (44–66)	0.808 (0.685–0.891)	0.00 (0.00–70.7)	0.00 (0.00–2.62)
L2	5	0.4–15.9/3rd tertile	81 (69–89)	69 (46–85)	–	0.00 (0.00–100)	0.00 (0.00–100)
LCR	3	0/3rd tertile	45.3 (19–73.4)	75.2(47.6–91.0)	–	0.00 (0.00–100)	0.00 (0.00–100)
LCR/L1/L2	3	5.3/3rd tertile	54.6 (26.1–80.4)	74.2 (45.8–90.7	–	0.00 (0.00–100)	0.00 (0.00–100)
L1 6367	5	3.1–10/3rd tertile	75 (62–85)	52 (37–67)	0.720 (0.665–0.769)	89.9 (68–99)	0.00 (0.00–1.45)
L1 6389	5	3.1–10/3rd tertile	82 (68–91)	48 (33–63)	0.834 (0.691–0.918)	98.9 (97–100)	0.18 90.00–6.58)
L1 5611	3	10.8/3rd tertile	–	–	0.830 (0.739–0.894)	96.4 (87–100)	0.22(0.05–9.24)
L1 6650	3	10.8/3rd tertile	–	–	0.779 (0.653–0.868)	97.8 (92–100)	0.30 (0.08–12.06)
L1 7145	5	10.8/3rd tertile	–	–	0.729 (0.649–0.796)	95.6 (88–100)	0.17 (0.06—1.55)
**≥CIN3/HSIL**							
L1	7	3.1–15.9	76 (66–84)	75 (57–87)	0.903 (0.804–0.955)	0.00 (0.00–58.6)	0.00 (0.00–0.77)

CIN: cervical intraepithelial neoplasia; WGA: Whole Genome Average.

Bivariate models applied wherever possible in preference. When not possible, univariate model were applied.

^a^ Number of studies and heterogeneity refers to pooled sensitivity meta-analyses, on occasion the number of studies for AUC meta-analyses was less due to less available data.

Restriction to studies at low risk of bias in the analyses of the HPV16 L1/L2 gene reduced heterogeneity (*I^2^*=81%); sensitivity was improved (80%) with no change in specificity (63%). Similarly, restriction to histopathology only studies increased sensitivity to 80·5%, while other analyses (including restriction to a 10% cut-off and pyrosequencing only techniques) did not change estimates (supplementary figures 13–17).

We also performed meta-analysis of AUC for CpG sites L15611, L16367, L16389, L16650 and L17145, for detection of CIN2+ and of L1 5611 for CIN3+. The greatest pooled estimated AUC for CIN2+ was observed at L16389 (0·83 (95%CI 0·69–0·92)) and L15611 (0·83 (95%CI 0·74–0·89)) (Table 4). Where meta-analysis of AUC was not possible, we plotted AUC values for diagnostic accuracy of methylation in predicting CIN2+ and observed a clustering of higher values in L1/L2 regions when compared to LCR (supplementary figure 18). This was similar for HPV18, 31, 33, 45 and 52, whilst for HPV31, the E1/E2 had greater AUC than the L1 gene (supplementary figure 19).

**Fig. 3:**
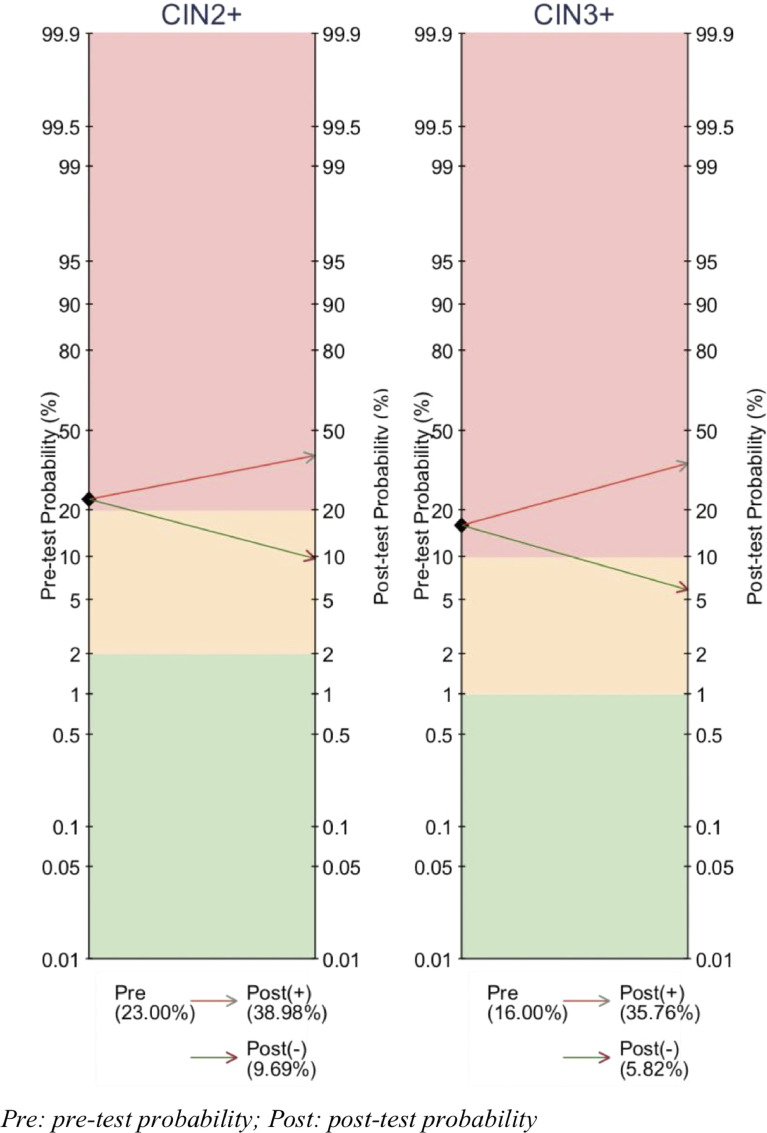
Pre- and post-test probability of HPV16 methylation testing for detection of (a) ≥CIN2 and (b) ≥CIN3 in HPV 16 positive women of a screening population. Colours represent clinical recommendations for degree of risk requiring referral to colposcopy where red = high risk, yellow = moderate risk and green = low risk. Pre-test probability of CIN2^+^ or CIN3^+^ estimated from a HPV16^+^ screening population PPV of CIN2^+^ or CIN3^+^.

We further compared the pre-test and post-test risk of CIN2 or worse and CIN3 or worse in a HPV16 positive screening population (Fig. 3). Using the highest performing genes and sites (pooled sensitivity 77% – specificity 64%), a positive methylation test increased the risk of CIN2 or worse from 23·0% to 39·0%, whilst a negative test result decreased the risk to 9·7%. Risk of CIN3 or worse in a HPV16+ woman with a positive methylation test rose from 16·0% to 35·8% whilst a negative test result decreased risk to 5·8%.

## 4 Discussion

### 4.1 Main results in context with published literature

We observed that the proportion of positive methylation and mean methylation levels increased with disease severity predominantly in L1 and L2 regions, but also in the E2 and E6 genes, while no correlation was present in the HPV16 LCR promoter/enhancer region. Pooled differences in the mean methylation levels and odds ratios for high methylation in ≥CIN2/HSIL vs. ≤CIN1/LSIL were greatest for HPV16 L1 gene followed by the L2. HPV16 L1/L2 genes had overall the best diagnostic accuracy in detecting CIN2 or worse. When applied in a pre- and post-test probability model with a 23% risk of CIN2 in HPV16 positive women, the risk of CIN2 or worse increased to 39% if methylation positive and decreased to 10%, if negative. Half of studies had moderate to high risk of bias according to QUADAS-2 criteria for diagnostic test accuracy. The heterogeneity was overall high for most analyses. We performed a series of sensitivity analyses to explore possible causes of heterogeneity however, residual heterogeneity remained high.

The published literature to date on HPV methylation in cervical disease has explored various HPV types, genes and adjacent CpG sites with conflicting results. The majority of studies explore HPV16 and consistently reports higher methylation levels with increasing disease severity for L1/L2, in line with our pooled results. Although human DNA methylation of promoter/enhancer regions have been previously associated with increased tumorigenesis in other cancers [61], the HPV LCR region revealed no correlation with disease severity in our analysis. The evidence in individual studies has been conflicting, with 15 studies reporting higher methylation in the LCR region in high-grade disease, while 6 studies observed lower methylation [37,[49], [50], [51]. The biological processes leading to variation in HPV CpG site performance are not well understood. The L1 and L2 genes code for late viral capsid proteins, particularly important for viral transmission during the early stages of infection. Possibly these genes are subsequently silenced, allowing prioritisation of E6/E7 oncogene expression for carcinogenesis [62]. Studies examining the whole HPV genome have so far been limited but suggest a global increase in methylation by disease grade except for the LCR [6,41]. As sequencing costs decrease, whole genome methylation analysis could prove of great benefit in determining CpG sites of best discrimination, avoiding bias and improving understanding of the variation in HPV methylation across the genome. Downstream functional studies would also be helpful for further mechanistic understanding of the role of HPV methylation in cervical precancer progression.

The detection of women at high risk of CIN2 or worse has been a great challenge for population-based screening. The introduction of the hrHPV DNA test in screening offers improved protection against invasive cervical cancer when compared with cytology [3] and is expected to replace cytology globally. HPV testing will inevitably increase the number of women that test positive at screening, while the optimal triage test to accurately select those warranting colposcopy remains unclear. Optimising detection, while minimising over-intervention in women at low risk, has major benefits. In many countries like the UK, reflex cytology will be used due to ease and existing infrastructure, although its sensitivity in the detection of CIN3 or worse, is at best 53–75% with a specificity of 78–86% [63], [64], [65]. Further molecular markers have been proposed to improve the specificity and NPV, although evidence on superiority of any one of these is lacking. P16/Ki-67 immunostaining appears to improve specificity but requires cytology reporting and highly-trained staff with additional costs [66]. HPV genotyping has also been suggested to increase specificity (70–71%) [67], with the advantage of a streamlined, automated HPV workflow and potential for self-sampling, but suffers from the inability to distinguish between transient and persistent viral infection and therefore has a low PPV (13–29%), that is likely to fall further with the drop of HPV prevalence in vaccinated cohorts [68]. HPV mRNA tests compare well to HPV genotyping assays, with a high sensitivity but no apparent increase in specificity [69] while E6/E7 protein assays have low sensitivity but may improve specificity over HPV genotyping and cytology [70].

The diagnostic accuracy of HPV16 L1/L2 methylation in the detection of CIN2 or worse compares well to other proposed triage markers (sensitivity = 77%, specificity = 64%, AUC = 0·73) with the highest pooled AUC for a single CpG site at HPV16 L16389 (0·83). HPV methylation has several practical advantages given that analysis can be streamlined in the same pipeline with that of hrHPV testing, allowing for future automation and potentially self-sampling. HPV methylation also performed well compared to host methylation for the detection of CIN2 or worse (sensitivity = 69–74%; specificity = 66–76%) [11].

There were sufficient data to meta-analyse sensitivity and specificity for two individual HPV16 CpG sites which performed well compared to combined markers. We were further able to perform pooled analysis for AUC values for 5 HPV16 CpG sites. We found the greatest AUC at L15611 and L16389 (0·83), outperforming AUC pooled estimates for all other combined markers including L1/L2 and individual genes L1 and L2. Pooled AUC estimates for L15611 and 6389 are also greater than the best performing estimates published for combined host and viral methylation markers for detection of CIN2 or worse in UK screening populations (AUC = 0·78, sensitivity = 74% (95%CI 59–85), specificity = 65% (95%CI 60–70%)) [43]. Continued genome-wide exploration of HPV genotypes should determine the most accurate CpG sites alone or in combination that will yield greater discrimination. Next-generation sequencing methylation assays could allow for accurate whole genome coverage, while also improving consistency of methylation testing. Eventual HPV methylation tests however should be targeted to sequencing the best performing CpG sites which will facilitate low-cost, high-throughput automation.

To date cervical screening targeted the detection of high-grade pre-invasive disease. Modern molecular markers have the potential to advance this beyond the current status quo and provide information on the true carcinogenic potential of hrHPV infections irrespective of histological disease grade, allowing prediction of women that are likely to have progressive or regressive infection and/or disease [71]. As local cervical treatment increases reproductive morbidity, ability to avoid unnecessary treatments has major benefits [72]. Several studies explored the value of methylation in predicting HPV persistence or development of high-grade CIN. One of the largest longitudinal cohorts from Guanacaste, Costa Rica [7], demonstrated that high methylation in L1/L2 of 72 pre-diagnostic specimens was associated with increased risk of future CIN2+, with an OR of up to 9·3 (95%CI 2·3–45·1). Niyazi et al. [48] found similar results for L1 methylation in 145 women infected with HPV16, with an OR of up to 9·9 (95%CI 3·6–27·4). Longitudinal data could not be meta-analysed in our study.

### 4.2 Strengths and limitations

This is the first systematic review and meta-analysis to appraise all published literature exploring the use of HPV methylation in cervical disease. We included 44 studies and over 8819 hrHPV positive women and explored data for all HPV subtypes, genes and CpG sites (including previously unpublished data) and provide a robust quality assessment of this literature. We performed meta-analytical pooling for a number of outcomes, including measures of positive methylation rate and mean methylation according to disease severity, along with odd ratios and diagnostic accuracy meta-analyses to determine test accuracy in detecting high-grade CIN in HPV positive women. To this end we have brought together a complex body of literature and present pooled estimates of methylation data by genotype, gene and CpG site, with corresponding risk of bias and heterogeneity assessments. We further calculated the pre- and post-test probability of high-grade CIN, providing a translational and understandable tool for policy makers, clinicians and patients to inform about risk.

There were however some limitations. Half of the studies were at moderate or high risk of bias and there was substantial heterogeneity for most analyses. The included studies used different eligibility criteria, sequencing tests, sampling techniques, and reference verification standards; many did not adhere to diagnostic test accuracy study principles including unbiased patient selection, investigator blinding to the reference test or avoidance of differential verification bias [13]. We performed a series of sensitivity analyses to explore heterogeneity. Restricting to studies at low risk of bias, or using histopathology for verification, or exfoliative cells only, reduced heterogeneity in analyses of mean methylation and ORs (≥CIN2/HSIL vs. ≤CIN1/LSIL). Restricting to pyrosequencing and NGS alone, did not. To further assess whether the transformation of median to means and imputation of SD values introduced bias, we performed sensitivity analysis to exclude imputed values, with a similar direction of effect observed. Most studies explored L1 and L2 genes that increased statistical power for these comparisons, and repeat assessment of selected CpG sites could have led to confirmation bias. Overall there appears to be a clear association between increasing HPV methylation with cervical disease grade, however large variation in effect sizes exists and residual heterogeneity is yet to be fully explained. The heterogeneity was overall high for most analyses. Although heterogeneity is known to be higher in meta-analyses of continuous rather than those of binary data [73] there are very likely other underlying reasons for the observed large heterogeneity. Methylation testing is still under development and not fully standardised. Furthermore, only a few studies assessed reproducibility by duplicate and triplicate analysis of experimental test results. Different studies have used different methylation techniques and reference standards. There was insufficient evidence to evaluate whether environmental factors, such as smoking [74], modifies HPV methylation levels to cause heterogeneity; this would be an important research direction for the future. Additionally, small study effects were found to be present where investigated (Egger's *P* < 0.01) and although overall we could not formally test all analyses, similar bias of smaller studies showing more extreme estimates might still be present and explain some of the interstudy heterogeneity. We now need larger studies and a collaborative approach, which uses a standardised methodological approach and cut-off, in order to accurately determine the strength of this association.

The substantial heterogeneity noted in the diagnostic test accuracy (DTA) analyses did not improve when restricting to good quality studies. This is likely due to studies using different cut-off points for positivity and exploring various CpG sites, resultantly pooled point estimates should be interpreted with caution. In our analysis, we aimed to explore this: where more than one cut-off was provided, we used the most overlapping cut-off thresholds between studies and performed a sensitivity analysis restricting to studies using 10% mean methylation as a cut-off. We used averages of CpG sites by gene but also analysed separately studies presenting single CpG sites where data were sufficient. The overall sensitivity and specificity did not differ greatly in either analysis, although AUC values for single CpG sites performed better than combined gene averages. Sensitivity analyses were small, and it is likely there was not sufficient power to adequately detect cut-off and CpG effects. Although not previously discussed, methylation ‘classifiers’ have attempted to bring the best performing CpG sites to a single marker and are currently under validation [43]. Future research should assess whether weighted combination of several best performing CpG sites has the potential to further improve the diagnostic accuracy. Following this it will be crucial to determine the best cut-off for an eventual diagnostic test, which our review suggests may vary according to the included CpG sites. It should be noted that a cut-off of 0% should not be used, to avoid confusion with failed assays. Once cut-offs and CpG sites have been established, robust and blinded diagnostic test accuracy studies are needed to reduce risk of bias in methylation studies and determine the true chances of false positives and negatives, including in including in populations of different geographical regions and ethnicities.

Despite these limitations, this meta-analysis provides the most comprehensive review and critical appraisal of the existing literature with stringent quality assessment of the methodological validity of existing studies; this information on the best performing techniques, cut-offs, genes and CPG sites should inform future design of diagnostic accuracy studies, including standardisation of assays. For translation to clinical practise we urgently need larger and higher quality evidence. To provide this, a collaborative and standardised research approach, with consideration of data deposition on publicly available repositories, should be encouraged in the field.

We calculated pre- and post-test probabilities based on the rates of high-grade CIN in HPV16 positive women irrespective of cytology as an example of a screening population. We used the best performing sites and genes (HPV16 L1/L2) and saw that methylation added good additional discrimination between high and low-grade disease, although pre-test probabilities of HPV16 positive women were high. Multiplexed methylation assays combining CpGs across all hrHPV types are currently under development [75], while multiplexing for HPV genotyping is becoming well established [76]. To increase the clinical utility of viral methylation testing, multiplexed methylation assays are needed to cover all hrHPV types and could be applied to triage women screening as hrHPV positive, who have not been genotyped. In this scenario, pre-test probabilities would be lower [18] and the value of methylation testing would increase. Our estimates represent the most accurate and conservative estimate of how future multiplexed assays, including all HPV genotypes, could perform for triage of hrHPV positive women.

## 5 Conclusions

This study provides the most comprehensive summary of how different HPV methylation CpGs, genes and combinations perform. We demonstrated that HPV methylation correlates with disease severity and calculated the diagnostic accuracy of methylation for individual HPV genotypes in detecting high-grade CIN in HPV positive women. These results demonstrate that viral methylation may be a useful epigenetic marker for cervical cancer prevention. It is anticipated that technical improvements and standardisation of protocols will further increase clinical accuracy of epigenetic markers. One of the major challenges is that viral methylation is type-specific and future tests should allow for a multitude of markers, targeting all hrHPV types, in one assay. Cut-offs need to be established and comparison of the performance of viral to host methylation will be needed, whilst their combination may further enhance accuracy.

Next-generation multiplex sequencing assays containing all HPV genotypes are under development and have the potential to allow rapid, automated and low-cost methylation testing. With the assumption that these will perform at least as well as the pooled diagnostic accuracy for HPV16 L1/L2, viral methylation has the potential to evolve to a useful candidate triage marker in women that test positive of hrHPV at primary screening, determining who will warrant further colposcopy. When such assays become available, large high-quality clinical trials in screening populations comparing various triage tools head-to head will be needed.

Our study provides important evidence that can inform future research directions and can enhance our understanding of the impact of epigenetic changes of the host and the virus in cervical disease and introduction of new molecular technologies to clinical practice. Future studies should explore epigenome-wide associations in the host, should validate reproducibility of high-quality multiplex assays for all HPV types, followed by clinical trials in screening populations. Exploration of methylation in longitudinal cohorts has the potential to also enlighten its possible predictive value in disease progression.

## Declaration of Competing Interest

No conflicts to declare.

## Appendix Supplementary materials

Image, application 1
