# Supplementary material for: The use of human papillomavirus DNA methylation in cervical intraepithelial neoplasia: A systematic review and meta-analysis
Source: eBioMedicine. 2019 Nov 12;50:246–59. doi: 10.1016/j.ebiom.2019.10.053 (PMC6921230; doi:10.1016/j.ebiom.2019.10.053)
Supplement: Supplementary file 1 [file mmc1.docx]

**Supplementary Methods**

**Glossary of terms**

≥CIN2/HSIL/high-grade cervical intraepithelial neoplasia (CIN): CIN grade 2, grade 3 or cervical cancer (histological definition) **^1^**; high-grade squamous intraepithelial lesion (cytological definition) ^2,3^

≤CIN1/LSIL/low-grade CIN: normal, atypical squamous cells of undetermined significance (ASCUS), CIN grade 1 (histological definition); low-grade squamous intraepithelial lesion (cytological definition).

CIN2+: CIN2 or worse

CIN3+: CIN 3 or worse

MD: Mean Difference, calculated by subtracting the smaller mean methylation from the larger.

Mean methylation level: the measured percentage of cytosines methylated at a particular CpG site out of the total number of cytosines measured in a particular sample (range 0-100%).

Proportion methylation: the proportion of samples where methylation was measured as present at a particular CpG site, out of the total number of samples measured.

Odds Ratio (OR) for methylation: the odds of high-grade disease being present, given high methylation at a particular region, at a chosen cut-off.

Diagnostic test accuracy for methylation: the accuracy of high methylation, at a particular region and chosen cut-off, in detecting high-grade disease.

**Selection criteria and Search Strategy**

With the following search criteria, studies were identified by two independent reviewers (SB, AM) and discrepancies resolved by a third author (IK) from MEDLINE, EMBASE and CENTRAL:

1. Uterine Cervical Neoplasms/

2. Cervical Intraepithelial Neoplasia/

3. CIN*.mp.

4. exp Uterine Cervical Dysplasia/

5. (cervi* adj5 (cancer* or tumor* or tumour* or malignan* or neoplas* or carcinoma* or adenocarcinoma* or precancer* or pre-cancer* or dysplasia or squamous or CIN* or HSIL or LSIL or ASCUS)).mp.

6. 1 or 2 or 3 or 4 or 5

7. exp papillomaviridae/

8. exp Papillomavirus Infections/

9. (HPV* or hpv* or human papilloma* or papilloma*).mp.

10. 7 or 8 or 9

11. exp methylation/

12. exp Epigenesis, Genetic/

13. Epigenomics/

14. (methylome* or epigenetic* or epigenome*).mp.

15. (bisulphite* or bisulfite* or pyrosequenc* or methylation* or mass spectrometry or mass-spectrometry or EpiTYPER).mp.

16. (next generation sequencing or NGS or high throughput sequencing or illumina array* or illumina sequenc* or solexa).mp.

17. (microsphere-based suspension array* or microsphere-based suspension array* or luminex Cmap or luminex C-map or luminex C map).mp.

18. (suspension adj5 (array* or technology*)).mp.

20. 11 or 12 or 13 or 14 or 15 or 16 or 17 or 18

21. 6 and 10 and 20

22. (animals not (humans and animals)).sh.

23. 21 not 22

Metaregister, Physicians Data Query, www.controlled-trials.com/rct, www.clinicaltrials.gov and www.cancer.gov/clinicaltrials were searched for ongoing studies. To identify studies which might have been missed during the electronic search, the citation lists of included studies were hand-searched and experts in the field, including directors of UK cancer and colposcopy registries, contacted to identify further reports of studies. We further checked the proceedings of the following relevant conferences:

- Annual Meeting of the British Society of Colposcopy and Cervical Pathology.
- Annual Meeting of the International Federation of Cervical Pathology and Colposcopy.
- EUROGIN.
- European Federation of Colposcopy Congresses.
- Annual Meeting of the American Society of Colposcopy and Cervical Pathology.

**Outcome Measures**

a) proportion of samples methylated at a CpG and/or gene (samples methylated/samples methylated plus samples unmethylated) b) percentage mean or median methylation at a CpG and/or gene (0-100% methylation) c) mean difference (MD) in methylation (mean methylation in high-grade disease minus mean methylation in low-grade disease) d) odds-ratio (OR) of high-grade vs. low-grade disease for high methylation

**Quality Assessment of studies**

We used a modified QUADAS-2 tool in line with the Cochrane recommendation for quality assessment and risk of bias in diagnostic test accuracy studies. We modified this allow evaluation of all studies including experimental and proof of principle studies, as previously described by Arbyn et al (Lancet Oncology, 2014).^4^ In the tailored QUADAS-2 criteria^5,6^, we assessed four domains: A) Patient selection - P1: acceptable enrolment method, P2: acceptable inclusion criteria and inappropriate exclusions avoided. B) Index test - I1: acceptable sample material (LBC, cervical swab, cervical tissue), I2: acceptable methylation test giving quantitative results (Pyrosequencing, EpiTYPER, Next generation sequencing, Luminex) C) Reference test - R1: acceptable reference test (histology confirmation of grade for CIN and cancer, at least cytology confirmation of grade for normal and ascus), R2: masking of methylation analysis to reference test; D) Flow and timing - F1: acceptable interval between index test and reference standard; F2: differential verification avoided; F3: withdrawals and uninterpretable results explained. We excluded ‘concerns regarding applicability’ from the assessment as the studies were observational, non- randomised and none assessed diagnostic test accuracy of HPV methylation as a diagnostic test in a cervical screening population.

Subdomains were assessed according to QUADAS-2 guidance by answering yes, no or unclear (where unclear relates to insufficient data for assessment). A summary risk of bias score was calculated and was used for sensitivity analysis. The four domains were classified as high, moderate or low risk of bias after summarising the results of the subdomains.

**Data extraction & Missing Data**

From each included study, two reviewers (SB, AM) extracted data on study design and setting, index test, sample material, HPV type, CpG sites analysed, reference standard, outcome measures and risk of bias using a predefined spreadsheet. Data were extracted for different HPV types separately and for individual CpG sites where available. Where data were missing (i.e. individual CpG sites data), we contacted the authors and collated additional data for a number of studies. ^7-11^ Where only medians were available we estimated means and the relevant standard deviation (SD) using the Wan et al. method.^12^ Where an SD was not provided or could not be calculated, we used the Furukawa et al imputation method.^13^ Where additional data were not provided by authors and graphs were of high quality, we used the WebPlotDigitizer ^14^ to estimate data points from graphs. National centre for biotechnology information (NCBI) reference genomes were used to locate CpG sites for all HPV types. ^15^

**Statistical Analysis**

We compared the proportions of methylated samples for ≥CIN2/HSIL and ≤CIN1/LSIL in a meta-analysis using the variance-stabilising Freeman-Tukey double arcsine transformation approach. In order to define methylation positivity, we used the author defined cut-off throughout. When several studies presented duplicate data from the same cohort,^8,10,16,17^ the study with the largest sample size and lowest risk of bias that presented data on the primary and secondary outcomes, was selected for inclusion. We assessed between-study heterogeneity across all meta-analyses using the Cochran’s Q test, a visual inspection of forest plots, and the I^2^ statistic along a 95% CI.^18-20^ We estimated the between-study variance using the restricted maximum likelihood approach in R and the DerSimonian and Laird method in Stata.

The details for methods of each meta-analysis were as follows:

Meta-analysis on proportions of methylated samples: Proportion of samples methylated at a CpG and/or gene was defined as: N samples methylated/ N samples methylated plus N samples unmethylated presented by studies. We used the individual author definition of methylated samples. Where proportions were provided per CpG site, we totalled proportion methylation per gene. Where a study only presented methylation data for a group of samples, this was. divided by the total number of samples to estimate the proportion of samples methylated. Where a study provided percentages of samples methylated only the number of samples methylated was calculated by multiplying the percentage methylation by total number of samples, therefore in some cases this was not an integer. Where studies presented results for multiple CpG sites per gene, the mean result for the gene was meta-analysed. We compared the proportions of methylated samples using the variance-stabilising Freeman-Tukey double arcsine transformation approach^21-23^ . We performed secondary analyses in more defined clinical groups to include normal, LSIL, HSIL and invasive cervical cancer (ICC).

Meta-analysis on mean methylation levels and mean difference: Mean/Median methylation levels were determined by percentage mean or median methylation at a CpG and/or gene, from 0-100% methylation presented by studies. In the meta-analysis of means, where methylation levels were averaged by gene, standard deviations (SD) were averaged according to standard recommendations ^24^. Mean difference values were calculated by subtracting Mean difference values in studies were calculated by subtracting mean methylation values from low-grade disease samples from high-grade disease samples, then individual CpG level data was averaged to give mean methylation levels by gene. Meta-analysis of MD was conducted on data averaged by gene and disease grade groups, assuming a normal distribution.

Odds ratios (OR) meta-analysis: Odds ratios were determined as presented by individual studies - where the proportion of methylated samples in a high-grade disease group was compared to proportion of methylated samples in a low-grade disease group. When calculated from mean/median methylation, this was according to a chosen cut-off as determined by the study. When a study did not present an odd ratio, but a proportion of samples methylated were presented for high-grade vs. low grade disease, we calculated the underlying odds ratio with a corresponding 95% CI. For studies not reporting odds ratios but reporting means and SDs we calculated the standardised mean differences, which then were transformed to odds ratio that could be combined with existing study-specific odds ratio pooled analyses ^18^.

Diagnostic test accuracy meta-analysis: For diagnostic accuracy meta-analysis, we used the true positive (TP), true negative (TN), false negative (FN) and false positive (FP), where available. When not available, these data were calculated from the total number of samples, sensitivity, specificity, positive predictive value (PPV), negative predictive value (NPV). We used author defined cut-off thresholds throughout, where cut-off thresholds varied, the most overlapping thresholds were chosen, and we used a single threshold from each study in the meta-analysis. Where multiple CpGs were tested, we aimed to combine the most commonly analysed CpGs. Where possible, average data for the whole gene were chosen, otherwise the best performing CPG site was selected. Data were combined to calculate pooled estimates using two models. Where possible a bivariate model was applied in STATA using the midas routine^25^, taking the correlation between true-positive and false-positive rates and between-study variability into account. Where data were insufficient to perform a bivariate analysis, we analysed sensitivity and specificity separately using the logit transformation in a univariate analysis using the metafor R package ^26^. In the bivariate mixed-effects binary regression model that was used in the diagnostic test accuracy studies, the DerSimonian and Laird estimator was used to estimate the between-study variance ^27^. Whereas in each univariate meta-analysis, the restricted maximum likelihood method was used. ^28,29^ A 95% CI for the between-study variance was calculated using the Q-profile approach ^30^. Pretest–posttest probability plots decision thresholds were based on best performing CpG combinations and benchmark risk levels of HPV16 positivity as reported across studies in Europe (2% and 20% for CIN2 or worse, 1% and 10% for CIN3 or worse, respectively). ^31^

All analyses were performed in R v3.6.1 and R studio Version 1.2.5001^32^ and STATA v14.^33^

Sensitivity analyses

**MD, OR, DTA:** a) studies at low risk of bias b) histopathology as reference test c) pyrosequencing as index test d) cervical exfoliated cells (CEC) as sample material.

**MD alone:** we additionally excluded imputed SD values for sensitivity analysis.

**OR alone:** we also excluded the transformed OR values from continuous data.

**DTA alone**: we also limited to those studies using a cut-off limited to 10% mean methylation.

The full research protocol can be found at Open science Framework.^34^

**PRISMA Research Checklist**

| **Section/topic** | **#** | **Checklist item** | **Reported on page #** |
| --- | --- | --- | --- |
| **TITLE** | | |  |
| Title | 1 | Identify the report as a systematic review, meta-analysis, or both. | 1 |
| **ABSTRACT** | | |  |
| Structured summary | 2 | Provide a structured summary including, as applicable: background; objectives; data sources; study eligibility criteria, participants, and interventions; study appraisal and synthesis methods; results; limitations; conclusions and implications of key findings; systematic review registration number. | 2 |
| **INTRODUCTION** | | |  |
| Rationale | 3 | Describe the rationale for the review in the context of what is already known. | 4 |
| Objectives | 4 | Provide an explicit statement of questions being addressed with reference to participants, interventions, comparisons, outcomes, and study design (PICOS). | 4 |
| **METHODS** | | |  |
| Protocol and registration | 5 | Indicate if a review protocol exists, if and where it can be accessed (e.g., Web address), and, if available, provide registration information including registration number. | 7 |
| Eligibility criteria | 6 | Specify study characteristics (e.g., PICOS, length of follow-up) and report characteristics (e.g., years considered, language, publication status) used as criteria for eligibility, giving rationale. | 5 |
| Information sources | 7 | Describe all information sources (e.g., databases with dates of coverage, contact with study authors to identify additional studies) in the search and date last searched. | 5 |
| Search | 8 | Present full electronic search strategy for at least one database, including any limits used, such that it could be repeated. | 5 (refers to supplement) |
| Study selection | 9 | State the process for selecting studies (i.e., screening, eligibility, included in systematic review, and, if applicable, included in the meta-analysis). | 5 |
| Data collection process | 10 | Describe method of data extraction from reports (e.g., piloted forms, independently, in duplicate) and any processes for obtaining and confirming data from investigators. | 5 |
| Data items | 11 | List and define all variables for which data were sought (e.g., PICOS, funding sources) and any assumptions and simplifications made. | 6 |
| Risk of bias in individual studies | 12 | Describe methods used for assessing risk of bias of individual studies (including specification of whether this was done at the study or outcome level), and how this information is to be used in any data synthesis. | 5 |
| Summary measures | 13 | State the principal summary measures (e.g., risk ratio, difference in means). | 6 |
| Synthesis of results | 14 | Describe the methods of handling data and combining results of studies, if done, including measures of consistency (e.g., I^2^) for each meta-analysis. | 6 |
| **Section/topic** | **#** | **Checklist item** | **Reported on page #** |
| Risk of bias across studies | 15 | Specify any assessment of risk of bias that may affect the cumulative evidence (e.g., publication bias, selective reporting within studies). | 6 |
| Additional analyses | 16 | Describe methods of additional analyses (e.g., sensitivity or subgroup analyses, meta-regression), if done, indicating which were pre-specified. | 6-7 |
| **RESULTS** | | |  |
| Study selection | 17 | Give numbers of studies screened, assessed for eligibility, and included in the review, with reasons for exclusions at each stage, ideally with a flow diagram. | 7 |
| Study characteristics | 18 | For each study, present characteristics for which data were extracted (e.g., study size, PICOS, follow-up period) and provide the citations. | 7 |
| Risk of bias within studies | 19 | Present data on risk of bias of each study and, if available, any outcome level assessment (see item 12). | 7 (refers to supplement) |
| Results of individual studies | 20 | For all outcomes considered (benefits or harms), present, for each study: (a) simple summary data for each intervention group (b) effect estimates and confidence intervals, ideally with a forest plot. | 8-10 |
| Synthesis of results | 21 | Present results of each meta-analysis done, including confidence intervals and measures of consistency. | 8-10 |
| Risk of bias across studies | 22 | Present results of any assessment of risk of bias across studies (see Item 15). | 7 |
| Additional analysis | 23 | Give results of additional analyses, if done (e.g., sensitivity or subgroup analyses, meta-regression [see Item 16]). | 8-10 |
| **DISCUSSION** | | |  |
| Summary of evidence | 24 | Summarize the main findings including the strength of evidence for each main outcome; consider their relevance to key groups (e.g., healthcare providers, users, and policy makers). | 10 |
| Limitations | 25 | Discuss limitations at study and outcome level (e.g., risk of bias), and at review-level (e.g., incomplete retrieval of identified research, reporting bias). | 12 |
| Conclusions | 26 | Provide a general interpretation of the results in the context of other evidence, and implications for future research. | 14 |
| **FUNDING** | | |  |
| Funding | 27 | Describe sources of funding for the systematic review and other support (e.g., supply of data); role of funders for the systematic review. | 15 |

From: Moher D, Liberati A, Tetzlaff J, Altman DG, The PRISMA Group (2009). Preferred Reporting Items for Systematic Reviews and Meta-Analyses: The PRISMA Statement. PLoS Med 6(7): e1000097. doi:10.1371/journal.pmed1000097

For more information, visit: **www.prisma-statement.org**.

**Supplement Table 1**. Characteristics of included studies.

| **Author, year** | **Country** | **Population** | **Material** | **Method (Index Test)** | **HPV subtype** | **CpG Sites** | **Sample (N)** | **Reference Standard** | **Outcome Measures** |
| --- | --- | --- | --- | --- | --- | --- | --- | --- | --- |
| Badal 2003 | New Mexico, Brazil | HPV16+ screening population | CEC (N), FFPE biopsy tissue (CIN, ICC) | MSRE | 16 | LCR and E2BS (11 CpGs) | 81: N (25), CIN1 (13), CIN3 (10), ICC (33) | C (N) & H (CIN & ICC) | Proportion of cases methylated per grade |
| Bhattacharjee 2006 | India | Married women attending family planning and cancer referral centre | CEC (N), biopsy tissue (ICC) | MSRE | 16 | LCR, E6 | 72: N (15), ICC (57) | C (N) & H (ICC) | Proportion of cases methylated per grade |
| Brandsma 2014 | USA, Senegal | USA: HPV 16+ women attending screening, Senegal: never-before screened attending clinic | CEC (ND) | BS | 16 | E5, L1, L2 (12 CpGs) | 33: CIN1 (16), CIN2 (7), CIN3 (5), ICC (5) | C & H (all) | Mean and Median percentage methylation per CpG site, Diagnostic accuracy of methylation in predicting CIN2, CIN3, ICC, ≥CIN2 |
| Brentnall 2014 | England | Women with abnormal cytology referred to colposcopy | CEC (all) | Pyro | 16, 18, 31 | L1, L2, LCR | 1493: ≤CIN1 (937), CIN2/3 (556) | C (all) & H (clinically indicated) | Proportion of cases methylated per grade, Mean percentage methylation per CpG site, Diagnostic accuracy of methylation in predicting CIN2/3 |
| Brentnall 2015 | England | Women with abnormal cytology referred to colposcopy | CEC (CIN) | Pyro | 33 | L1, L2 | 1493: ≤CIN1 (937), CIN2/3 (556) | C (all) & H clinically indicated) | Proportion of cases methylated per grade, Diagnostic accuracy of methylation in predicting CIN2/3 |
| Bryant 2014 | Wales | HPV+ screening population and Cancers | CEC (N, Severe dyskaryosis) and tissue biopsy (ICC) | Pyro | 16 | LCR, L1, L2, E2 | 51: N (17), Severe dyskaryosis (20), ICC (24) | C (N/severe dyskaryosis) & H (ICC) | Median percentage methylation per CpG site, Diagnostic accuracy of methylation in predicting severe dyskaryosis* (*cancer not included) |
| Bryant 2015 | Wales | HPV16+ attending colposcopy | CEC (N, CIN) | Pyro | 16 | E2, L1, L2 (12 CpGs) | 234: Cytology - N (22), Borderline (63), Mild (54), Moderate (39), Severe (52), Glandular neoplasia (2), unknown (2). (Histopathology (200) - N (21), CIN1 (34), CIN2 (51), CIN3 (92), ACC in situ (2)) | C (all) & H (N=200/234) | Median percentage methylation per CpG site, Diagnostic accuracy of methylation in predicting ≥CIN2 and ≥CIN3 |
| Chaiwongkot 2013 | Thailand, Germany | Women with abnormal cytology referred to hospital | FFPE (CIN, ICC) | Pyro | 16 | LCR (10 CpGs) | 51: <CIN2/3 (18), ICC (33) | H (all) | Methylation levels for an individual HPV DNA molecule at multiple CpG Sites (methyl-haplotype), methylation levels in samples with integrated genomes vs. episomal |
| Chaiwongkot 2017 | India | HPV+ screening cohort | Cervical swab (all) | Pyro | 16 | L1 | 101: Normal (15), CIN1 (21), CIN2 (29) CIN3(4) ICC (32) | C&H (all) | Mean percentage methylation per CpG site |
| Clarke 2018 | USA | HPV+ screening population | CEC | NGS | 12 HPV types | L1 and L2 | 659: 360 Normal and ASCUS, 299 CIN3 and ICC (AIS) | C & H (all) | Median percentage methylation per CpG site. |
| Ding 2009 | Taiwan | HPV16+ with confirmed CIN and ICC | CEC (N, CIN), tissue biopsy (ICC) | BS | 16 | LCR | 53: LSIL (17), HSIL (21), ICC (15) | C & H (all) | Proportion of cases methylated per grade |
| Dutta 2015 | India | HPV16+ screening population and ICC referred to hospital | Cervical swabs (N, LSIL, HSIL) Fresh frozen tissue biopsy (ICC) | MSRE | 16 | LCR | 611: N (89), LSIL (4), HSIL (24), ICC (98) | C (CIN) & H (ICC) | Proportion of cases methylated per grade |
| Frimer 2015 | Costa Rica | HPV16+ screening population | CEC (all) | NGS | 16 | E6, E2, L1, L2 (21 CpGs) | 65: N (21), CIN3 (31), ICC (13), longitudinal (13) | C (all) & H (ND) | Mean percentage methylation per CpG site, mean percentage methylation in HPV persistence vs. clearance |
| Gasperov 2015 | Croatia | HPV 16+ screening population and confirmed ICC receiving surgical treatment | CEC (all) | MSP | 16, 18, human | LCR, L1 (19 CpGs) | 12 HPV 16+: N (4), LSIL (2) HSIL (4), ICC (2). 22 HPV18+: LSIL/CIN1 (9), HSIL/CIN2 (6), HSIL/CIN3 (6), ICC (1) | C (all) & H (ICC) | Proportion of cases methylated per grade |
| Hong 2008 | China | HPV 16+ screening population and confirmed ICC referred to hospital | CEC (all) | Pyro | 16 | LCR | 70: N (14), CIN1/2 (17), CIN3 (13), ICC (26) | C (N) & H (CIN, ICC) | Proportion of cases methylated per grade |
| Hsu 2017 | Taiwan | HPV+ screening cohort | CEC (all) | Pyro | 16, 18, 52, 58 | L1 | 147: N (28), CIN1 (45), CIN2 (13), CIN3/CIS (61) | C & H (all) | Mean percentage methylation per CpG site |
| Hublarova 2009 | Czech Republic | HPV 16 + screening population and ICC referred to hospital | CEC (all) | MSRE | 16 | LCR, E2BS | 141: N (21), CIN1 (8), <CIN2/3 (89), ICC (23) | C & H (all) | Proportion of cases methylated per grade |
| Jacquin 2013 | France | Not defined | CEC (all) | Pyro and HRM-PCR | 16 | LCR (E2BS1, E2BS2, SP1) | 119: N (37), LSIL (30), HSIL (35), ICC (17) | C (all) & H (clinically indicated) | Mean percentage methylation per CpG site, Proportion of cases methylated per grade |
| Kalantari 2014 | California | Consecutive patients attending colposcopy clinic | CEC (all) | BS | 16, 18, 31, 45 | L1, L2 | 63: N (6), ASCUS (9), LSIL/CIN1 (14), HSIL/<CIN2/3 (21), ICC (13) | C (all) & H (clinically indicated) | Proportion of cases methylated per grade (in triplicate), Diagnostic accuracy of methylation in predicting ICC |
| Kottaridi 2017 | Greece, England | HPV+ women attending gynaecology clinics | CEC (all) | Pyro | 16 | L1 (12 CpGs) | 150: N (12), CIN1 (23), CIN2 (45), CIN3 (61), ICC (9) | C & H (all) | Mean percentage methylation per CpG site, Diagnostic accuracy of methylation in predicting ≥CIN2, ≥CIN3 & ICC |
| Leung 2015 | Hong Kong | HPV+ screening population | Vaginal swabs/Paraffin blocks (N, ASCUS, LSIL, HSIL) and FFPE (ICC) | Pyro | 16, 18 | LCR (E2BS1,2 & 4) | 137: N (15), Low-grade CIN/ASCUS (28), High-grade CIN (42) ICC (52) | C & H (ND) | Mean percentage methylation per CpG site |
| Liu 2017 | USA | Women with CIN or ICC. Otherwise not defined | CEC and Fresh Frozen Tissue | NGS and Pyrosequencing | 13 HPV types | Whole genome | NGS 60: CIN1 (2), CIN2 (2), CIN3 (20), ICC (37). Pyro 111: (CIN3 (61), ICC (50)) | C & H (all) | Mean percentage methylation per CpG site Diagnostic Accuracy of methylation in predicting ICC |
| Lorincz 2013 | Wales | HPV16+ screening population | CEC (all) | Pyro | 16 | E6, L1, L2, LCR | Study 1 (association with disease grade) (82): <CIN (67), CIN2 (10), CIN3 (15). Study 2 (persistence): HPV persistence at 6-months (73) | C (all) & H (clinically indicated) | Mean percentage methylation per CpG site, Mean percentage methylation in HPV persistence vs. clearance at 6 months, Diagnostic accuracy of predicting CIN2/3 & persistence of HPV16 at 6-months |
| Lorincz 2016 | England | HPV+ screening population | CEC (all) | Pyro | 16, 18, 31, 33, human | L1, L2 | 99; HPV 16 (87): ≤CIN2 (66) ≥CIN2 (21) | C & H (all) | Diagnostic accuracy of methylation in predicting ≥CIN2, ≥CIN3 12 months from an abnormal smear |
| Louvanto 2015 | Canada | Cases: Abnormal cytology referred to colposcopy Controls: matched age controls attending screening at same time period. 12 additional HPV positive CIN1 samples from a separate study | CEC (all) | Pyro | 16 | L1 (2 CpGs) | 210: N (36), CIN1 (21), CIN2 (47), CIN3 (47), SCC (31), ADC (28) | C & H (all) | Methylation levels for an individual HPV DNA molecule at multiple CpG Sites (methyl-haplotype), Diagnostic accuracy of methylation in predicting ≥CIN2 |
| Marongiu 2014 | England | HPV16+ screening population | CEC (all) | Pyro (16), BS (18, 31, 45) | 16, 18, 31, 45 | LCR, E6, L2, L1. HPV16 (20 CpGs) HPV18 (29 CpGs) HPV31 (4 CpGs) HPV45 (12 CpGs) | 79 HPV16+, 18 HPV18+, 50 HPV31+, 18 HPV45+. (LSIL (67), HSIL (125)) | C (all) & H (approx. 20% of all samples) | Median percentage methylation per CpG site, Diagnostic accuracy of methylation in predicting HSIL |
| Mirabello 2012 | Costa Rica | HPV16+ screening population | Cervical swab (all) | Pyro | 16 | LCR, L1 (20 CpGs) | Study 1 (Diagnostic cohort) (99): HPV16 clearance <2 years (34), HPV16 persistence 2+ years (35), CIN3 (30). Study 2 (Pre-diagnostic cohort) (89): HPV16 clearance (34), HPV16 persistence (35), CIN3 (20) | C & H (clinically indicated) | Mean percentage methylation per CpG site, Mean percentage methylation in HPV persistence vs clearance |
| Mirabello 2013 | Costa Rica | HPV16+ screening population | Cervical swab (all) | Pyro | 16 | LCR, E6, E7, L1 (20 CpGs) | 149: N (92), ≥CIN2 (57). | C & H (clinically indicated) | Mean percentage methylation per CpG site |
| Mirabello 2015 | USA | HPV 16+ screening population and confirmed ICC referred to hospital | CEC (all) | Pyro and NGS | 16` | E6, E2, L2, L1 (18 CpGs) | 99: N (40) CIN3 (59) | C (all) & H (CIN2/CIN3/ICC) | Median percentage methylation per CpG site, Diagnostic accuracy of methylation in predicting CIN2-3 |
| Murakami 2013 | Japan | HPV+ screening population | CEC (all) | BS | 52, 58 | HPV52: LCR, L1 (7 CpGs). HPV58: LCR, L1 (6 CpGs) | HPV52+ (54): <CIN1 (8), CIN1 (9), CIN2 (15), CIN3 (22) HPV58+ (32): <CIN1 (1), CIN1 (10), CIN2 (9), CIN3 (12). Persistence HPV52+ 12-months (15): CIN1 (6) CIN2 (9) | C (all) & H (clinically indicated) | Proportion of cases methylated per grade |
| Niyazi 2017 | China | HPV+ women attending screening | CEC (all) | Pyro | 16 | L1 (13 CpGs) | 145: N (51) (transient HPV infection (32), persistent HPV infection at 12-months (21)), CIN 1 (21), CIN2-3 (33) ICC (38) | C & H (all) | Median percentage methylation per CpG site, Diagnostic accuracy of methylation in predicting ≥CIN2 and persistent HPV infection, Median percentage methylation in HPV infection persistence vs clearance |
| Patel 2012 | USA | HPV16+ screening population | CEC (all) | Pyro | 16 | LCR, E6, L1 (10 CpGs) | 89: N (15), ASCUS (36), LSIL (24), HSIL (12), AGC (2) | C (all) | Proportion of cases methylated per grade |
| Piyathilake 2011 | USA | HPV16+ screening population | CEC (all) | Pyro | 16 | LCR (6 CpGs) | 75: ≤CIN1 (45) ≥CIN2 (30) | C & H (all) | Mean percentage methylation per CpG site |
| Piyathilake 2014 | USA | HPV16+ screening population | CEC (all) | Pyro | 16 | LCR (6 CpGs) | 309: ≤CIN1 (225). CIN2 (34), CIN3 (50) | C (all) & H (clinically indicated) | Median percentage methylation per CpG site |
| Qiu 2015 (1) | China | Cytopathology samples sent to a university hospital | CEC (all) | MS-HRM | 16 | L1 | 114: CIN<2 (42) ≥CIN2 (72) | C (all) & H (clinically indicated) | Proportion of samples methylated per disease grade, Diagnostic accuracy of methylation in predicting ≥CIN2 |
| Qiu 2015 (2) | China | Cytopathology samples sent to a university hospital | CEC (all) | MS-HRM | 16 | L1 | 81: N (16), CIN1 (16), CIN2 (16), CIN3 (26), ICC (7) | C & H (all) | Proportion of samples methylated per disease grade, Diagnostic accuracy of methylation in predicting ≥CIN2 |
| Simanaviciene 2015 | Lithuania | Women with abnormal cytology or histology plus N controls from a screening population. | CEC (all) | BS | 16, 18, 51 | LCR, L1 (17 CpGs) | 157: N (29), CIN1 (10), CIN2 (17), CIN3/CIS (70), ICC (31) | C & H (all) | Proportion of cases methylated per grade |
| Snellenberg 2012 | Netherlands | HPV16+ screening population | CEC (N, CIN3) and FFPE (CIN3, ICC) | Luminex C-map | 16 | LCR (E2BS1, E2BS3, E2BS4) | 65: N (17) CIN3 (19), ICC (29) | C (N, CIN3) & H (all) | Median percentage methylation per CpG Site |
| Sun 2011 | USA | HPV16+ screening population | CEC (all) | Pyro and EpiTYPER | 16 | LCR, L1 | 85: N/LSIL/CIN1 (46) CIN2 (12) CIN3/ICC (27) | C (N, LSIL) & H (CIN1, CIN2, CIN3, ICC) | Proportion of cases methylated per grade |
| Vasilijevic 2014 | England | Women referred to colposcopy with mild dyskaryosis or worse | CEC (all) | Pyro | 18, 31, 33 | HPV18 (L2, L1, LCR, E6) HPV31 (L2, L1, LCR) HPV33 (L2, L1, LCR) | 205 HPV18+, 206 HPV31+, 117HPV33+: ≤CIN1 (302) vs. ≥CIN2 (226) | C (all) & H (clinically indicated & all in persistence study arm samples) | Median percentage methylation per CpG site |
| Wang 2017 | China | HPV+ women | CEC and Fresh Frozen Tissue | BS | 16 | L1 and LCR (18 CpGs) | 122: N/LSIL (37), HSIL (35), ICC (37) | C & H (all) | Proportion of cases methylated per grade and Diagnostic accuracy of methylation in predicting HSIL (AUC only) |
| Wentzensen 2012 | USA | HPV18, 31, 45+ women age >/21 attending screening and women age <30 with ASCUS | CEC (all) | Pyro | 18, 31, 45 | Whole genome (focusing on L1, L2 and E2 but >/1 site per gene) | 188: ≤CIN1/ASCUS (96), CIN3 (92) | C & H (all) | Median percentage methylation per CpG Site |
| Xi 2011 | USA | HPV16+ screening population | CEC (all) | BS | 16 | LCR (11 CpGs) | 211: ≤CIN1 (117), ≥CIN2 (94) | C & H (all) | Median percentage methylation per CpG Site |
| Zhang 2019 | China | Consecutive HPV16+ screening population | CEC (all) | Pyro | 16 | LCR (E2BS: 7 CpGs) | 43: N( 10), LSIL(15), HSIL/ICC (18), | C(N) & H(LSIL/HSIL/ICC) | Diagnostic accuracy of methylation in predicting HSIL+ |

**Supplementary Table 2**. Meta-analysis of proportion of HPV methylated samples for Normal vs ASCUS/CIN1/LSIL vs. CIN2/CIN3HSIL vs ICC. The grade was defined by histology; if this was not available, cytology was used.

| **Author, year** | **Cut-off** | **CpG** | **Study size (n)** | **Normal % (n/N)** | **CIN1/LSIL/ASCUS**  **% (n/N)** | **CIN2/CIN3/HSIL**  **% (n/N)** | **ICC % (n/N)** |
| --- | --- | --- | --- | --- | --- | --- | --- |
| **HPV16 L1** | | | | | | | |
| Qiu 2015 (1) | >10% | L1 (bp5602, 5608, 5611, 5617) | 114 | 5.9 (1/17) | 64 (16/25) | 90.2 (55/61) | 100 (11/11) |
| Qiu 2015 (2) | >10% | L1 (bp7136, 7145) | 81 | 6.3 (1/16) | 62.5 (10/16) | 88.1 (37/42) | 100 (7/7) |
| Simanaviciene 2015 | >0% | L1 (bp7136, 7145) | 157 | 3.4 (1/29) | 0 (0/10) | 20.7 (18/87) | 35.5 (11/31) |
| Gasperov 2014 | >0% | L1 (bp7091, 7136, 7145, 7145) | 12 | 75 (3/4) | 0 (0/2) | 0 (0/4) | 100 (2/2) |
| Kalantari 2014 | >0% | L1/L2 (bp 5602-7270) | 63 | 8.3 (1/6) | 13.0 (3/23) | 33.3 (7/21) | 83.1 (10/13) |
| Wang 2017 | >0% | L1 (bp 7089-7268) | 100 | - | 16.6 (5/30)* | 34.3 (12/35) | 65.7 (23/35) |
| **Total N – n/N** |  |  | **527** | **7/72** | **34/106** | **129/250** | **64/99** |
| **Pooled Estimate (95% CI)** |  |  |  | **13.9 (0.3-36.8)** | **31.1 (6.2-62.1)** | **49.4 (20.0-79.0)** | **76.7 (53.6-94.7)** |
| I^2^ (95%CI) |  |  |  | 65.88 (0.00-96.98) | 82.5 (49.4-97.7) | 95.1 (86.8-99.2) | 75.1 (52.1-93.3) |
| τ^2^ (95%CI) |  |  |  | 0.04 (0.00-0.72) | 0.08 (0.02-0.70) | 0.12 (0.04-0.79) | 75.5 (34.1-95.3) |
| Q-test (P-value) |  |  |  | 10.53 (0.03) | 24.56 (<0.001) | 122.43 (<0.001) | 21.49 (<0.001) |
|  | | | | | | | |
| **HPV 16 L2** | | | | | | | |
| Kalantari 2014 | >0% | L1/L2 (bp 5602-7270) | 63 | 8.3 (1/6) | 13.1 (3/23) | 33.3 (7/21) | 83.1 (10/13) |
| **Total N – n/N** |  |  | **63** | **1/6** | **3/23** | **7/21** | **10/13** |
|  | | | | | | | |
| **HPV16 LCR** | | | | | | | |
| Badal 2003 | >0% | 5' LCR, Enh, E6 promoter (bp ND) | 81 | 52 (13/25) | 23 (3/13) | 20 (2/10) | 6.1 (2/33) |
| Bhattacharjee 2006 | >0% | 5'LCR (bp7289-7540) | 72 | 40 (6/15) | - | - | 54.4 (31/57) |
| Ding 2009 | >0% | 5'LCR, Enh, E6 promoter (bp7426-58) | 53 | - | 5.9 (1/17) | 33.3 (7/21) | 53.3 (8/15) |
| Dutta 2015 | >0% | Enh (bp7535-7694), E6 promoter | 215 | 44.6 (40/89) | 25 (1/4) | 37.5 (9/24) | 45.6 (45/98) |
| Hublarova 2009 | >0% | E6 promoter (bp7851-7559) | 141 | 81 (17/21) | 62.5 (5/8) | 31.5 (28/89) | 43.4 (10/23) |
| Gasperov 2015 | >0% | 3'LCR, Enh, Promoter (bp7091-58) | 12 | 47 (2/4) | 50 (1/2) | 25 (1/4) | 43.4 (1/2) |
| Simanaviciene 2015 | >0% | 5'LCR, Enh, Promoter (bp7270-58) | 157 | 4.3 (1/29) | 0 (0/10) | 21.8 (19/87) | 29 (9/31) |
| Snellenberg 2012 | >0% | E2BS1 (bp 7370-7383) | 65 | 12 (2/17) | N/A | 21.1 (4/19) | 93 (27/29) |
| Hong 2008 | >0% | Enh, Prom (bp7676-58) | 70 | 71.4 (10/14) | 29.4 (5/17) | 46.2 (6/13) | 84.6 (22/26) |
| Wang 2017 | >0% | 3'L1, 5'LCR, Enh, Promoter (bp 7089-58) | 101 | - | 8.8 (3/34)* | 18.2 (6/33) | 47 (16/34) |
| **Total N – n/N** |  |  | **967** | **91/214** | **19/105** | **82/300** | **171/348** |
| **Pooled Estimate (95% CI)** |  |  |  | 41.8 (21.0-64.1) | 22.6 (7.1-42.0) | 26.6 (20.7-32.7) | 50.9(31.8-69.8) |
| I^2^ (95%CI) |  |  |  | 88.1 (71.2-97.0) | 53.5 (0.0-89.9) | 11.1 (0.0-71.3) | 90.5 (78.5-97.0) |
| τ^2^ (95%CI) |  |  |  | 0.08 (0.03-0.34) | 0.03 (0.00-0.22) | 0.00 (0.00-0.00) | 0.07 (0.03-0.24) |
| Q-test (P-value) |  |  |  | 54.6 (<0.001) | 12.46(0.05) | 7.45 (0.45) | 82.0 (<0.001) |
|  | | | | | | | |
| **HPV18 L1/L2** | | | | | | | |
| Simanaviciene 2015 | >0% | L1 (bp6916-7122) | 21 | 12.5 (1/8) | 33.3 (1/3) | 50 (2/4) | 100 (6/6) |
| Gasperov 2015 | >0% | L1 (bp7017-7140) | 22 | - | 100 (9/9) | 91.7 (11/12) | 100 (1/1) |
| Kalantari 2014 | >0% | L1/L2 (bp5602-7270) | 14 | 0 (0/2) | 20 (1/5) | 100 (2/2) | 80 (4/5) |
| **Total N – n/N** |  |  | **57** | **1/10** | **11/17** | **15/18** | **11/12** |
| **Pooled Estimate (95% CI)** |  |  |  | **10.5 (0.0-42.4)** | **56.8 (5.7-99.7)** | **83.6 (51.4-100)** | **91.4 (61.8-100)** |
| I^2^ (95%CI) |  |  |  | 0.0 (0.0-98.9) | 76.6 (19.9-99.3) | 0.0 (0.0-69.1) | 0.00 (0.00-91.9) |
| τ^2^ (95%CI) |  |  |  | 0.0 (0.00-4.53) | 0.14 (0.01-6.38) | 0.0 (0.00-0.03) | 0.0 (0.0-0.59) |
| Q-test (P-value) |  |  |  | 0.09 (0.77) | 9.74 (0.01) | 0.56 (0.91) | 0.63 (0.73) |
|  | | | | | | | |
| **HPV18 LCR** | | | | | | | |
| Simanaviciene 2015 | >0% | LCR (LCR5', Prom, Enh) | 21 | 0 (0/8) | 0 (0/3) | 0 (0/4) | 22.2 (1.3/6) |
| **Total N – n/N** |  |  | **21** | **0/8** | **0/3** | **0/4** | **1.3/6** |
|  | | | | | | | |
| **HPV 31** | | | | | | | |
| Kalantari 2014 | >0% | L1/L2 (bp5518-5692) | 15 | 0 (0/1) | 0 (0/3) | 0 (0/3) | 48.6 (1.9/4) |
| **Total N – n/N** |  |  | **15** | **0/1** | **0/3** | **0/3** | **1.9/4** |
|  | | | | | | | |
| **HPV 45 L1/L2** | | | | | | | |
| Kalantari 2014 | >0% | L1/L2 (bp4795-7135) | 12 | 0 (0/1) | 0 (0/1) | 75.9 (2.3/3) | 94.4 (5.2/6) |
| **Total N – n/N** |  |  | **12** | **0/1** | **0/1** | **2.3/3** | **5.2/6** |
|  | | | | | | | |
| **HPV 52 L1** | | | | | | | |
| Murakami 2013 | >0% | L1 (bp5606-7120) | 54 | 15 (2.6/17)** | 34 (5.1/15) | 52 (11.4/22) | - |
| **Total N – n/N** |  |  | **54** | **2.6/17** | **5.1/15** | **11.4/22** |  |
|  | | | | | | | |
| **HPV 52 LCR** | | | | | | | |
| Murakami 2013 | >0% | LCR (LCR5', Prom, Enh) | 54 | 2.5 (0.4/17) | 1.3 (0.2/15) | 3.2 (0.7/22) | - |
| **Total N – n/N** |  |  | **54** | **0.4/17** | **0.2/15** | **0.7/22** |  |
|  | | | | | | | |
| **HPV 58** | | | | | | | |
| Murakami 2013 | >0% | L1 (bp5606-7119) | 41 | 12 (2.4/20)** | 38 (3.4/9) | 61 (7.3/12) | - |
| **Total N – n/N** |  |  | **41** | **2.4/20** | **3.4/9** | **7.3/12** |  |
|  | | | | | | | |
| **HPV 58** | | | | | | | |
| Murakami 2013 | >0% | LCR (LCR5', Prom, Enh) | 41 | 0 (0/20)** | 0 (0/9) | 0 (0/12) | - |
| **Total N – n/N** |  |  | **41** | **0/20** | **0/9** | **0/12** |  |

ASCUS: abnormal squamous cells of undetermined significance; Bp: base pair; CIN: cervical intraepithelial neoplasia; Enh: enhancer region; HSIL: high-grade squamous intraepithelial lesion; ICC: invasive cervical carcinoma (squamous or adenocarcinoma; LCR: long control region; LSIL: low-grade squamous intraepithelial lesion; N: total number of samples; n: number of samples methylated; ND: not defined; Prom: promoter region; Q-test: Cochrane Q test.

* Normal & LSIL

** Cervicitis & CIN1

**Supplement Table 3.** Sensitivity meta-analysis of proportion of HPV methylated samples for different genotypes and genes defined by histology alone for: A) ≤CIN1 vs. ≥CIN2; B) Normal vs. CIN1 vs. CIN2/CIN3 vs. ICC.

| **Author, year** | **Cut-off (+)** | **CpG** | **Total N** | **≤CIN1 vs. ≥CIN2** | | **Normal vs. CIN1 vs. CIN2/CIN3 vs ICC** | | | | |
| --- | --- | --- | --- | --- | --- | --- | --- | --- | --- | --- |
|  |  |  |  | **≤CIN1 (n/N)** | **≥CIN2 (n/N)** | **Normal % (n/N)** | **CIN1 % (n/N)** | **CIN2 % (n/N)** | **CIN3 % (n/N)** | **ICC % (n/N)** |
| **HPV16 L1** |  |  |  |  |  |  |  |  |  |  |
| Qiu 2015 (1) | >10% | L1 (bp5602, 5608, 5611, 5617) | 114 | 40.5 (17/42) | 91.6 (66/72) | 5.9 (1/17) | 64 (16/25) | 86.2 (25/29) | 93.8 (30/32) | 100 (11/11) |
| Qiu 2015 (2) | >10% | L1 (bp7136, 7145) | 81 | 34.3 (11/32) | 89.8 (44/49) | 6.3 (1/16) | 62.5 (10/16) | 81.3 (13/16) | 92.3 (24/26) | 100 (7/7) |
| Simanaviciene 2015 | >0% | L1 (bp7136, 7145) | 157 | 2.6 (1/39) | 24.6 (29/118) | 3.4 (1/29) | 0 (0/10) | 5.9 (1/17) | 24.3 (17/70) | 35.5 (11/31) |
| Gasperov 2015 | >0% | L1 (bp7091, 7136, 7145, 7145) | 12 | - | - | 75 (3/4) | - | - | - | 100 (2/2) |
| Kalantari 2014 | >0% | L1/L2 (bp 5602-7270) | 63 | - | - | 8.3 (1/6) | - | - | - | 83.1 (10/13) |
| Wang 2017 | >0% | L1 (bp 7089-7268) | 100 | - | 50 (35/70) | - | - | - | - | 65.7 (23/35) |
| **Total N – n/N** |  |  | **527** | **29/113** | **209/309** | **8/72** | **26/51** | **39/62** | **71/128** | **64/99** |
| **Pooled Estimate (95% CI)** |  |  |  | **24.1 (5.3-50.0)** | **71.5 (24.8-99.8)** | **13.9 (0.3-36.8)** | **42.4 (6.9-82.6)** | **57.5 (6.9-99.3)** | **73.3 (24.1-100)** | **76.7 (53.6-94.7)** |
| I^2^ (95%CI) |  |  |  | 89.4 (66.9-99.2) | 97.98 (92.6-99.9) | 65.88 (0.00-96.98) | 88.2 (54.0-99.7) | 94.9 (81.0-99.9) | 96.7 (88-99.9) | 70.4(24.4-92.1) |
| τ^2^ (95%CI) |  |  |  | 0.064 (0.015-98.7) | 0.174 (0.04-6.97) | 0.04 (0.00-0.72) | 0.11 (0.02-5.20) | 0.23 (0.05-7.58) | 0.19 (0.05-7.58) | 0.04 (0.01-0.21) |
| Q-test (P-value) |  |  |  | 29.08 (<0.001) | 120.6 (<0.001) | 10.53 (0.03) | 14.41(<0.001) | 37.61 (<0.001) | 74.31 (<0.001) | 21.49 (<0.001) |
|  | | | | | | | | | | |
| **HPV 16 L2** | | | | | | | | | | |
| Kalantari 2014 | >0% | L1/L2 (bp 5602-7270) | 63 | - | - | 8.3 (1/6) | - | - | - | 83.1 (10/13) |
| **Total N – n/N** |  |  | **63** | **-** | **-** | **1/6** | **-** | **-** | **-** | **10/13** |
| **HPV16 LCR** |  |  |  |  |  |  |  |  |  |  |
| Badal 2003 | >0% | 5' LCR, Enh, E6 Prom (bp ND) | 81 | 42.1 (16/38) | 9.3 (4/43) | 52 (13/25) | 23.1 (3/13) | - | 20 (2/10) | 6.1 (2/33) |
| Bhattacharjee 2006 | >0% | 5'LCR (bp7289-7540) | 72 | 4 (6/15) | 54.4 (31/57) | 40 (6/15) | - | - | - | 54.4 (31/57) |
| Ding 2009 | >0% | 5'LCR, Enh, E6 Prom (bp7426-58) | 53 | - | - | - | - | - | - | 53.3 (8/15) |
| Dutta 2015 | >0% | Enh (bp7535-7694), E6 Prom | 215 | - | - | 44.6 (40/89) | - | - | - | 45.6 (45/98) |
| Hublarova 2009 | >0% | E6 Prom (bp7851-7559) | 141 | 81 (17/21) | 35.8 (43/120) | 81 (17/21) | 62.5 (5/8) | 31.5 (28/89)* | - | 43.4 (10/23) |
| Gasperov 2015 | >0% | 3'LCR, Enh, Prom (bp7091-58) | 12 | - | - | 47 (2/4) | - | - | - | 43.4 (1/2) |
| Simanaviciene 2015 | >0% | 5'LCR, Enh, Prom (bp7270-58) | 157 | 2.7 (1/39) | 23.7 (28/118) | 4.3 (1/29) | 2.5 (0/10) | 11.8 (2/17) | 24 (17/70) | 29 (9/31) |
| Snellenberg 2012 | >0% | E2BS1 (bp 7370-7383) | 65 | 11.8 (2/17) | 64.6 (31/48) | 12 (2/17) | - | - | 21 (4/19) | 93 (27/29) |
| Hong 2008 | >0% | Enh, Prom (bp7676-58) | 70 | 48.4 (15/31) | 71.8 (28/39) | 71.4 (10/14) | 29.4 (5/17) (CIN1-2) | - | 46.2 (6/13) | 84.6 (22/26) |
| Wang 2017 | >0% | 3'L1, 5'LCR, Enh, Prom (bp 7089-58) | 101 | 8.8 (3/34) | 32.8 (22/67) | - | - | - | - | 47 (16/34) |
| **Total N – n/N** |  |  | **967** | **60/195** | **246/492** | **91/214** | **13/48** | **30/106** | **29/112** | **171/348** |
| **Pooled Estimate (95% CI)** |  |  |  | **35.4 (15.5-58.3)** | **36.3 (22.0-51.9)** |  | **28.4 (8.5-53.0)** | **11.8 (0.3-32.3)** | **27.8 (21.5-34.6)** | **51.6 (33.2-69.8)** |
| I^2^ (95%CI) |  |  |  | 92.8 (82.1-98.5) | 92.3 (80.8-98.5) | 88.1 (71.2-97.0) | 61.4 (0.0_97.5) | N/A | 0.0 (0.0-90.2) | 89.4 (75.8-96.7) |
| τ^2^ (95%CI) |  |  |  | 0.08 (0.03-0.44) | 0.04 (0.01-0.22) | 0.08 (0.03-0.34) | 0.03 (0.00-0.85) | N/A | 0.00 (0.00-0.07) | 0.07 (0.02-0.23) |
| Q-test (P-value) |  |  |  | 71.67 (<0.001) | 54.3 (<0.001) | 54.6 (<0.001) | 7.49 (0.06) | N/A | 3.20 (0.53) | 69.69 (<0.001) |
|  | | | | | | | | | | |
| **HPV18 L1/L2** | | | | | | | | | | |
| Simanaviciene 2015 | >0% | L1 (bp6916-7122) | 21 | 18.2 (2/11) | 80 (8/10) | 12.5 (1/8) | 33.3 (1/3) | 0 (0/2) | 100 (2/2) | 100 (6/6) |
| Gasperov 2015 | >0% | L1 (bp7017-7140) | 22 | - | - | - | - | - | - | 100 (1/1) |
| Kalantari 2014 | >0% | L1/L2 (bp5602-7270) | 14 | - | - | 0 (0/2) | - | - | - | 80 (4/5) |
| **Total N – n/N** |  |  | **57** | **2/11** | **8/10** | **1/10** | **1/3** | **0/2** | **2/2** | **11/12** |
| **Pooled Estimate (95% CI)** |  |  |  |  |  | **10.5 (0.00-42.4)** |  |  |  | **91.4 (62.1-100)** |
| I^2^ (95%CI) |  |  |  |  |  | 0.0 (0.0-98.9) |  |  |  | 0.00 (0.00-91.9) |
| τ^2^ (95%CI) |  |  |  |  |  | 0.0 (0.00-4.53) |  |  |  | 0.0 (0.0-0.59) |
| Q-test (P-value) |  |  |  |  |  | 0.09 (0.77) |  |  |  | 0.83 (0.73) |
|  | | | | | | | | | | |
| **HPV18 LCR** | | | | | | | | | | |
| Simanaviciene 2015 | >0% | LCR (LCR5', Enh, Prom) | 21 | 0 (0/13) | 20 (2/8) | 0 (0/8) | 0 (0/3) | 0 (0/2) | 16.7 (0.3/2) | 22.2 (1.3/6) |
| **Total N – n/N** |  |  | **21** | **0/13** | **2/8** | **0/8** | **0/3** | **0/2** | **0.3/2** | **1.3/6** |
|  | | | | | | | | | | |
| **HPV 31** | | | | | | | | | | |
| Kalantari 2014 | >0% | L1/L2 (bp5518-5692) | 15 | - | - | 0 (0/1) | - | - | - | 48.6 (1.9/4) |
| **Total N – n/N** |  |  | **15** |  |  | **0/1** |  |  |  | **1.9/4** |
|  | | | | | | | | | | |
| **HPV 45 L1/L2** | | | | | | | | | | |
| Kalantari 2014 | >0% | L1/L2 (bp4795-7135) | 12 | - | - | 0 (0/1) | - | - | - | 94.4 (5.2/6) |
| **Total N – n/N** |  |  | **12** |  |  | **0/1** |  |  |  | **5.2/6** |
|  | | | | | | | | | | |
| **HPV 52 L1** | | | | | | | | | | |
| Murakami 2013 | >0% | L1 (bp5606-7120) | 54 | - | - | - | 15 (2.6/17)** | 34 (5.1/15) | 52 (11.4/22) | - |
| **Total N – n/N** |  |  | **54** |  |  |  | **2.6/17** | **5.1/15** | **11.4/22** |  |
|  | | | | | | | | | | |
| **HPV 52 LCR** | | | | | | | | | | |
| Murakami 2013 | >0% | LCR (LCR5', Prom, Enh) | 54 | - | - |  | 2.5 (0.4/17)** | 1.3 (0.2/15) | 3.2 (7/22) | - |
| **Total N – n/N** |  |  | **54** |  |  |  | **0.4/17** | **0.2/15** | **7/22** |  |
|  | | | | | | | | | | |
| **HPV 58** | | | | | | | | | | |
| Murakami 2013 | >0% | L1 (bp5606-7119) | 41 | - | - |  | 12 (2.4/20)** | 38 (2.4/9) | 61 (7.3/12) | - |
| **Total N – n/N** |  |  | **41** |  |  |  | **2.4/20** | **2.4/9** | **7.3/12** |  |
|  | | | | | | | | | | |
| **HPV 58** | | | | | | | | | | |
| Murakami 2013 | >0% | LCR (LCR5', Prom, Enh) | 41 | - | - |  | 0 (0/20)** | 0 (0/9) | 0 (0/12) | - |
| **Total N – n/N** |  |  | **41** |  |  |  | **0/20** | **0/9** | **0/12** |  |

ASCUS: abnormal squamous cells of undetermined significance; Bp: base pair; CIN1-3: cervical intraepithelial neoplasia grades 1 to 3; Enh: enhancer region HSIL: high-grade squamous intraepithelial lesions; ICC: invasive cervical carcinoma (squamous or adenocarcinoma; LCR: long control region; LSIL: low-grade squamous intraepithelial lesion; N: total number of samples; n: number of samples methylated; ND: not defined; Prom: promoter region; Q-test: Cochrane Q test.

* CIN2-3

** Cervicitis & CIN1

**Supplement Table 4.** Sensitivity analysis of the pooled mean methylation levels for HPV16 only for studies using histology alone for reference for different disease grades (normal, CIN1, CIN2, CIN3, ICC) and averaged per gene, with corresponding heterogeneity estimates.

| **Disease Grade*/Gene** | **Studies (n)** | **Mean** | **CI_L** | **CI_H** | **τ^2^** | **CI_L**  **(τ^2^)** | **CI_H**  **(τ^2^)** | **I^2^ (%)** | **CI_L**  **(I^2^)** | **CI_H**  **(I^2^)** | **Q-test** | **P-value^+^** |
| --- | --- | --- | --- | --- | --- | --- | --- | --- | --- | --- | --- | --- |
| **Normal** | | | | | | | | | | | | |
| E1 | 1 | 3.90 | 2.13 | 5.67 | N/A | N/A | N/A | N/A | N/A | N/A | N/A | N/A |
| E2 | 4 | 4.62 | 1.30 | 7.93 | 12.51 | 3.11 | 125.12 | 92.86 | 76.37 | 99.24 | 28.27 | <0.001 |
| E5 | 1 | 13.95 | 9.84 | 18.06 | N/A | N/A | N/A | N/A | N/A | N/A | N/A | N/A |
| E6 | 2 | 1.14 | 0.84 | 1.45 | 0.00 | 0.00 | 27.22 | 0.00 | 0.00 | 99.66 | 4.12 | 0.13 |
| E7 | 1 | 5.10 | 4.00 | 6.21 | N/A | N/A | N/A | N/A | N/A | N/A | N/A | N/A |
| L1 | 9 | 9.75 | 4.87 | 14.64 | 50.78 | 20.63 | 188.88 | 98.64 | 96.72 | 99.63 | 270.12 | <0.001 |
| L2 | 2 | 7.51 | 6.15 | 8.87 | 0.00 | 0.00 | 39.63 | 0.00 | 0.00 | 96.46 | 1.54 | 0.46 |
| LCR | 5 | 4.07 | 2.17 | 5.97 | 3.99 | 1.10 | 34.75 | 92.15 | 76.46 | 99.03 | 64.34 | <0.001 |
| **CIN1** | | | | | | | | | | | | |
| L1 | 4 | 8.47 | 2.71 | 13.23 | 27.23 | 8.86 | 240.28 | 95.99 | 88.43 | 99,52 | 76.78 | <0.001 |
| **CIN2** | | | | | | | | | | | | |
| L1 | 5 | 10.60 | 3.17 | 18.02 | 70.50 | 24.59 | 584.87 | 98.66 | 96.25 | 999.84 | 341.11 | <0.001 |
| **CIN3** | | | | | | | | | | | | |
| E1 | 1 | 7.93 | 2.64 | 13.23 | N/A | N/A | N/A | N/A | N/A | N/A | N/A | N/A |
| E2 | 3 | 11.09 | 2.79 | 19.40 | 42.45 | 5.06 | 424.48 | 80.59 | 33.10 | 97.65 | 12.50 | 0.002 |
| E5 | 1 | 26.60 | 17.71 | 35.49 | N/A | N/A | N/A | N/A | N/A | N/A | N/A | N/A |
| E6 | 2 | 3.30 | 1.54 | 5.07 | 0.00 | 0.00 | 100.00 | 0.00 | 0.00 | 95.67 | 0.62 | 0.432 |
| E7 | 1 | 12.90 | 5.81 | 19.99 | N/A | N/A | N/A | N/A | N/A | N/A | N/A | N/A |
| L1 | 6 | 19.95 | 10.62 | 29.28 | 113.45 | 36.22 | 965.38 | 97.21 | 91.76 | 99.66 | 144.71 | <0.001 |
| L2 | 2 | 15.10 | 8.40 | 21.79 | 16.03 | 0.00 | 160.25 | 64.01 | 0.00 | 94.68 | 2.78 | 0.096 |
| LCR | 2 | 31.31 | -21.03 | 83.64 | 1423.20 | 281.03 | 14232.00 | 99.80 | 99.01 | 99.98 | 505.28 | <0.001 |
| **ICC** | | | | | | | | | | | | |
| L1 | 5 | 42.03 | 25.05 | 59.01 | 59.50 | 117.88 | 3060.69 | 96.90 | 91.15 | 99.63 | 150.15 | <0.001 |
| LCR | 3 | 21.86 | 6.55 | 37.17 | 179.43 | 45.95 | 1794.28 | 98.17 | 93.20 | 99.81 | 105.87 | <0.001 |

CI_L: confidence interval low: CI_H: confidence interval high; CIN: cervical intraepithelial neoplasia; LCR: long control region; Q-test: Cochrane Q test; SE: standard error.

^*^Pooled estimates of mean methylation level by gene performed according to histology confirmed disease grade, except in normal grade where cytology only was acceptable. ^+^P-value for Cochrane Q-test presented.

**Supplementary Table 5.** Sensitivity analysis of pooled difference in mean methylation level (MD) between ≤CIN1/LSIL vs. ≥CIN2/HSIL for HPV16 after exclusion of studies: with a low risk of bias only; used histology alone for reference; pyrosequencing only; exfoliated cervical cells only; where standard deviation was imputed.

| **Meta-analysis** | **Studies (n)** | **Mean Difference** | **CI_L** | **CI_H** | **τ^2^** | **CI_L**  **(τ^2^)** | **CI_H**  **(τ^2^)** | **I^2^ (%)** | **CI_L**  **(I^2^)** | **CI_H**  **(I^2^)** | **Q-test** | **P-value** |
| --- | --- | --- | --- | --- | --- | --- | --- | --- | --- | --- | --- | --- |
| **Main Analysis** |  |  |  |  |  |  |  |  |  |  |  |  |
| E1 | 1 | 4.03 | -1.55 | 9.61 | N/A | N/A | N/A | N/A | N/A | N/A | N/A | N/A |
| E2 | 4 | 8.84 | 0.22 | 17.47 | 67.46 | 14.69 | 674.62 | 88.01 | 61.51 | 98.66 | 24.04 | 0.00 |
| E5 | 1 | 12.65 | 2.86 | 22.44 | N/A | N/A | N/A | N/A | N/A | N/A | N/A | N/A |
| E6 | 3 | 2.45 | 0.49 | 4.42 | 0.53 | 0.00 | 100.00 | 13.38 | 0.00 | 96.67 | 2.50 | 0.29 |
| E7 | 2 | 7.72 | 3.74 | 11.70 | 0.00 | 0.00 | 100.00 | 0.00 | 0.00 | 91.14 | 0.02 | 0.88 |
| L1 | 12 | 11.29 | 6.46 | 16.13 | 63.36 | 27.19 | 196.15 | 92.97 | 85.01 | 97.61 | 124.39 | 0.00 |
| L2 | 5 | 5.62 | 1.82 | 9.41 | 14.53 | 2.35 | 145.25 | 88.01 | 54.30 | 98.66 | 17.74 | 0.00 |
| LCR | 6 | 1.55 | -0.28 | 3.38 | 3.55 | 0.38 | 33.52 | 77.14 | 26.61 | 96.96 | 16.14 | 0.01 |
|  | | | | | | | | | | | | |
| **Sensitivity Analyses** | | | | | | | | | | | | |
| **Low ROB** |  |  |  |  |  |  |  |  |  |  |  |  |
| L1 | 10 | 8.89 | 4.49 | 13.29 | 41.70 | 15.51 | 140.57 | 90.65 | 78.29 | 97.03 | 84.26 | 0.00 |
| L2 | 4 | 6.94 | 1.93 | 11.95 | 20.25 | 1.37 | 202.47 | 81.99 | 23.55 | 97.85 | 10.94 | 0.01 |
| LCR | 5 | 0.71 | -0.34 | 1.77 | 0.47 | 0.00 | 6.14 | 32.92 | 0.00 | 86.53 | 4.89 | 0.30 |
| **Histology only** | | | | | | | | | | | | |
| L1 | 7 | 8.89 | 4.49 | 13.29 | 41.70 | 15.51 | 140.57 | 90.65 | 78.29 | 97.03 | 84.26 | 0.00 |
| L2 | 2 | 6.94 | 1.93 | 11.95 | 20.25 | 1.37 | 202.47 | 81.99 | 23.55 | 97.85 | 10.94 | 0.01 |
| LCR | 1 | 0.71 | -0.34 | 1.77 | N/A | N/A | N/A | N/A | N/A | N/A | N/A | N/A |
| **Pyrosequencing only** | | | | | | | | | | | | |
| L1 | 7 | 8.89 | 4.49 | 13.29 | 41.70 | 15.51 | 140.57 | 90.65 | 78.29 | 97.03 | 84.26 | 0.00 |
| L2 | 2 | 6.94 | 1.93 | 11.95 | 20.25 | 1.37 | 202.47 | 81.99 | 23.55 | 97.85 | 10.94 | 0.01 |
| LCR | 1 | 0.71 | -0.34 | 1.77 | N/A | N/A | N/A | N/A | N/A | N/A | N/A | N/A |
| **Exfoliated cervical cells only** | | | | | | | | | | | | |
| L1 | 10 | 9.58 | 4.47 | 14.70 | 58.35 | 22.73 | 211.27 | 92.88 | 83.57 | 97.93 | 93.83 | 0.00 |
| L2 | 4 | 12.72 | -4.16 | 29.60 | 366.79 | 128.01 | 3118.43 | 99.49 | 98.54 | 99.94 | 219.23 | 0.00 |
| LCR | 4 | 1.84 | -1.23 | 4.91 | 7.71 | 1.15 | 100.00 | 80.40 | 38.05 | 98.16 | 15.86 | 0.00 |
| **Exclude SD imputed** | | | | | | | | | | | | |
| E1 | 1 | 4.03 | -1.55 | 9.61 | N/A | N/A | N/A | N/A | N/A | N/A | N/A | N/A |
| E2 | 2 | 8.15 | 3.26 | 13.04 | 2.28 | 0.00 | 100.00 | 18.00 | 0.00 | 90.60 | 1.22 | 0.27 |
| E5 | 1 | 12.65 | 2.86 | 22.44 | N/A | N/A | N/A | N/A | N/A | N/A | N/A | N/A |
| E6 | 2 | 3.15 | -0.17 | 6.47 | 3.43 | 0.00 | 100.00 | 54.41 | 0.00 | 97.21 | 2.19 | 0.14 |
| E7 | 2 | 7.72 | 3.74 | 11.70 | 0.00 | 0.00 | 100.00 | 0.00 | 0.00 | 91.14 | 0.02 | 0.88 |
| L1 | 5 | 9.53 | 2.88 | 16.18 | 47.58 | 11.10 | 448.04 | 93.18 | 76.11 | 99.23 | 27.47 | 0.00 |
| L2 | 4 | 6.19 | 0.63 | 11.76 | 27.61 | 4.21 | 276.11 | 93.78 | 69.72 | 99.34 | 16.19 | 0.00 |
| LCR | 2 | 1.21 | 0.47 | 1.96 | 0.00 | 0.00 | 21.06 | 0.00 | 0.00 | 93.81 | 0.02 | 0.90 |

CEC: exfoliated cervical cells; CI_L: confidence interval low: CI_H: confidence interval high; CIN: cervical intraepithelial neoplasia; LCR: long control region; MD: Mean difference. Pyro: pyrosequencing; Q-test: Cochrane Q test; RoGM: ratio of geometric means P-value: P-value of Cochran’s Q; SE: standard error.

**Supplementary Table 6.** Metanalysis of Odds Ratio of positive methylation (as defined by each study) for ≥CIN2/HSIL vs ≤CIN1/LSIL for all HPV genotypes and genes. Sensitivity analyses of HPV16 after exclusion of studies: with a low risk of bias only; used histology alone for reference; pyrosequencing only; where odd-ratio calculated from mean difference.

| **Meta-analysis** | **Studies (n)** | **OR** | **CI_L** | **CI_H** | **τ^2^** | **CI_L**  **(τ^2^)** | **CI_H**  **(τ^2^)** | **I^2^ (%)** | **CI_L**  **(I^2^)** | **CI_H**  **(I^2^)** | **z-test** | **P-value** |
| --- | --- | --- | --- | --- | --- | --- | --- | --- | --- | --- | --- | --- |
| **Main Analysis** | | | | | | | | | | | | |
| **HPV 16** | | | | | | | | | | | | |
| L1 | 22 | 5.48 | 3.53 | 8.49 | 0.79 | 0.34 | 1.77 | 85.63 | 72.05 | 93.06 | 7.60 | 2.931E-14 |
| L2 | 7 | 4.15 | 2.19 | 7.87 | 0.46 | 0.05 | 2.52 | 71.61 | 20.30 | 93.30 | 4.37 | 1.26E-05 |
| LCR | 19 | 1.71 | 0.96 | 3.03 | 1.25 | 0.58 | 3.64 | 89.68 | 80.26 | 96.20 | 1.82 | 6.82E-02 |
| E2 | 4 | 2.57 | 1.12 | 5.94 | 0.54 | 0.07 | 9.43 | 77.53 | 30.05 | 98.37 | 2.22 | 2.66E-02 |
| E6 | 4 | 2.13 | 1.44 | 3.16 | 0.00 | 0.00 | 5.23 | 0.00 | 0.00 | 96.81 | 3.79 | 1.52E-04 |
| **HPV18** |  |  |  |  |  |  |  |  |  |  |  |  |
| L1 | 7 | 8.31 | 2.94 | 23.51 | 1.00 | 0.09 | 10.87 | 61.82 | 12.21 | 94.61 | 3.99 | 6.538E-05 |
| **HPV31** |  |  |  |  |  |  |  |  |  |  |  |  |
| L1 | 5 | 4.48 | 3.03 | 6.62 | 0.00 | 0.00 | 1.93 | 0.00 | 0.00 | 86.80 | 7.50 | 5.396E-14 |
| **HPV52** |  |  |  |  |  |  |  |  |  |  |  |  |
| L1 | 2 | 6.51 | 2.57 | 16.46 | 0.00 | 0.00 | 0.00 | 0.00 | 0.00 | 0.00 | 3.95 | 7.672E-05 |
| **HPV58** |  |  |  |  |  |  |  |  |  |  |  |  |
| L1 | 2 | 6.20 | 2.45 | 15.69 | 0.00 | 0.00 | 79.78 | 0.00 | 0.00 | 99.34 | 3.85 | 1.173E-04 |
|  | | | | | | | | | | | | |
| **Sensitivity Analyses** | | | | | | | | | | | | |
| **HPV 16** | | | | | | | | | | | | |
| **Low ROB** | | | | | | | | | | | | |
| **L1** | 11 | 4.05 | 1.88 | 8.74 | 1.30 | 0.44 | 4.29 | 83.68 | 63.40 | 94.45 | 3.56 | 3.595E-04 |
| **Histology only** | | | | | | | | | | | | |
| L1 | 11 | 5.64 | 2.80 | 11.38 | 1.07 | 0.35 | 3.80 | 79.29 | 55.92 | 93.17 | 4.83 | 1.325E-06 |
| **Pyrosequencing only** | | | | | | | | | | | | |
| L1 | 14 | 3.09 | 1.68 | 5.69 | 1.06 | 0.42 | 3.18 | 90.69 | 79.44 | 96.69 | 3.62 | 2.887E-04 |
| **Exfoliated cervical cells only** | | | | | | | | | | | | |
| L1 | 18 | 3.72 | 2.18 | 6.36 | 0.95 | 0.37 | 2.26 | 87.08 | 72.23 | 94.13 | 4.82 | 1.511E-06 |
| **Exclude OR values calculated from MD** | | | | | | | | | | | | |
| L1 | 16 | 6.49 | 3.86 | 10.92 | 0.76 | 0.27 | 1.95 | 81.23 | 60.80 | 91.78 | 7.06 | 1.790E-12 |

CI_L: confidence interval low: CI_H: confidence interval high; CIN: cervical intraepithelial neoplasia; LCR: long control region; OR: odds ratio; P-value: P-value of OR: SE: standard error.

**Supplementary figure 1.** PRISMA flowchart


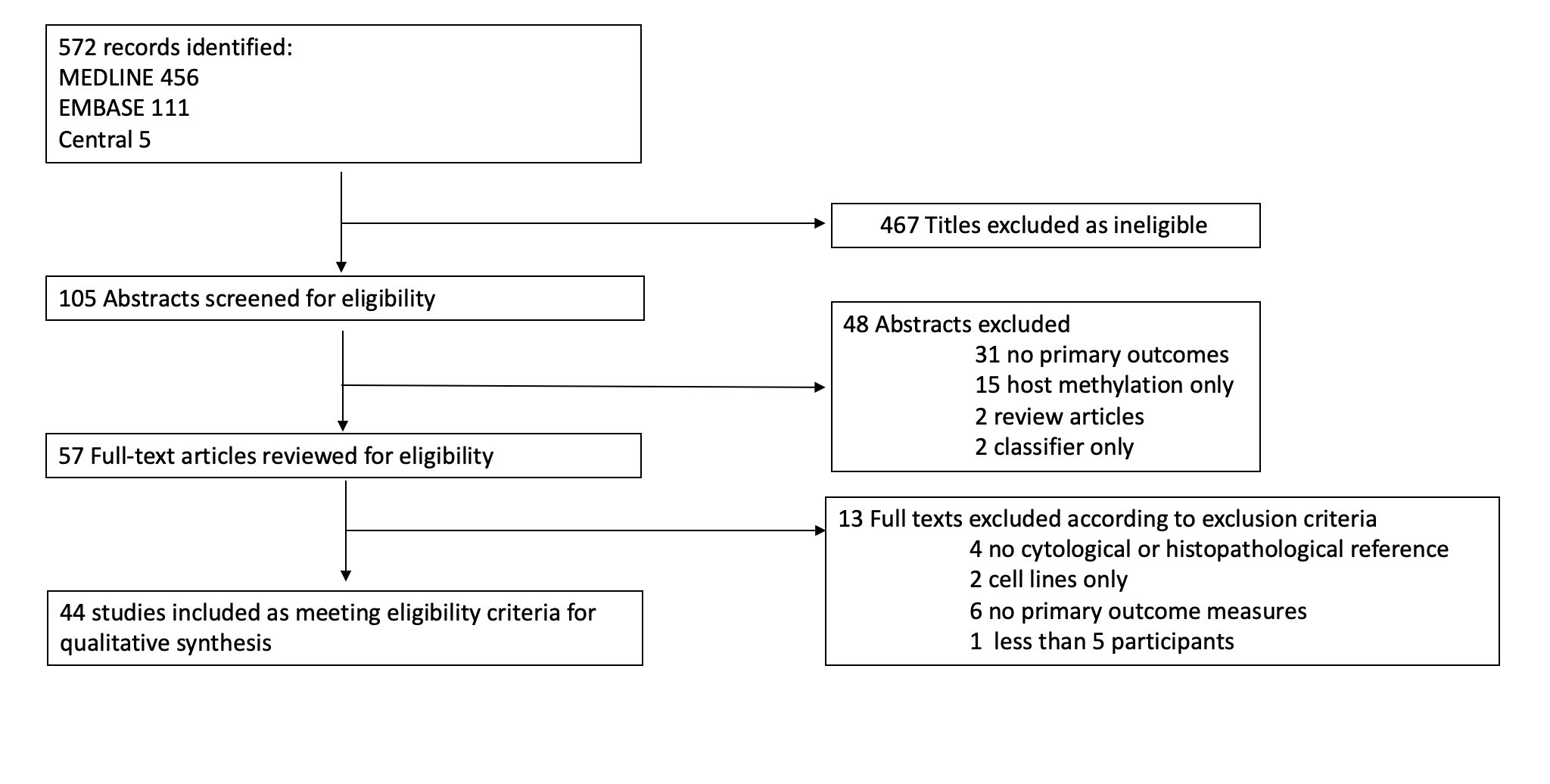


**Supplementary figure 2.** Funnel plot of publication bias assessment and small study effects

**
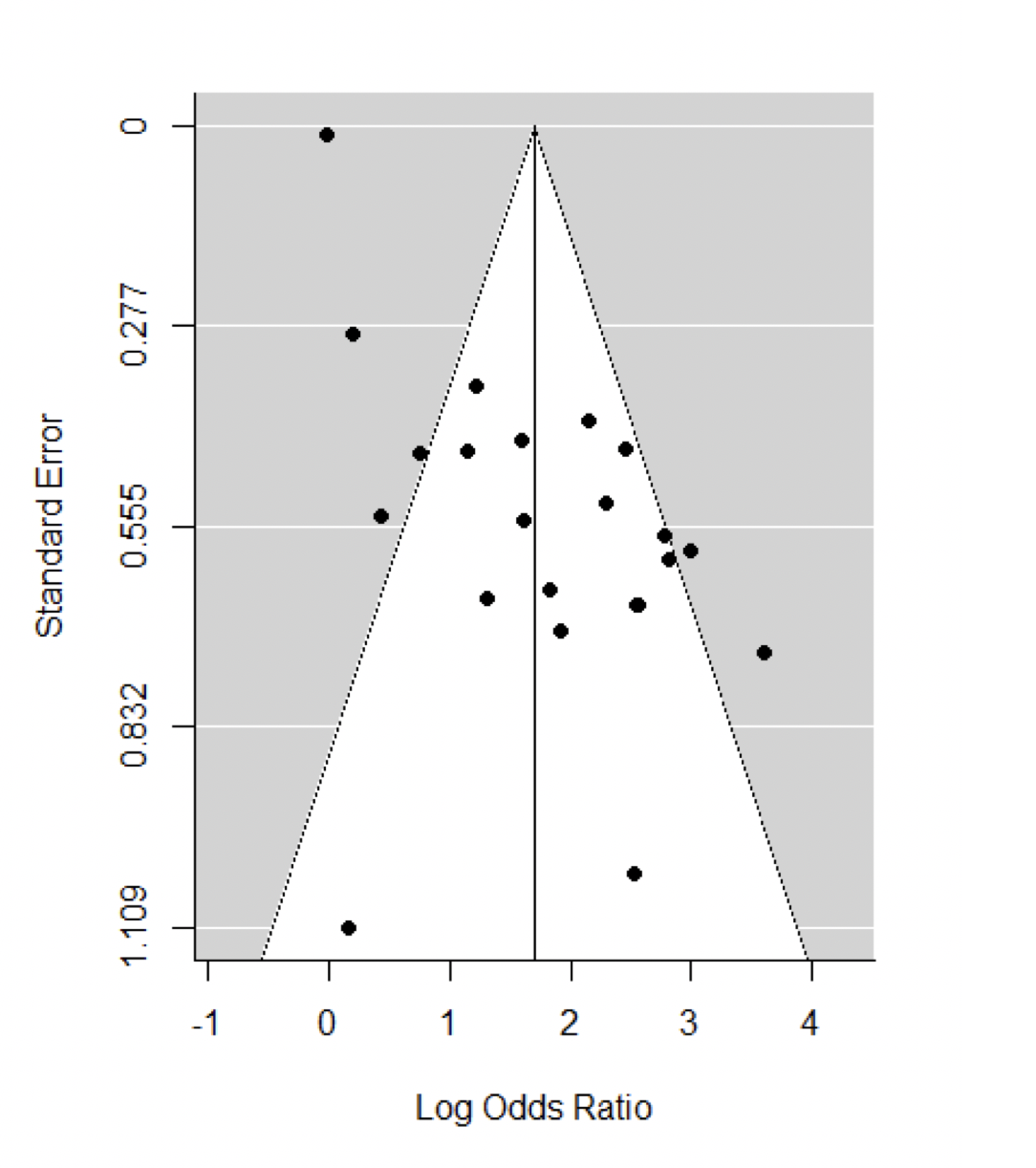
**


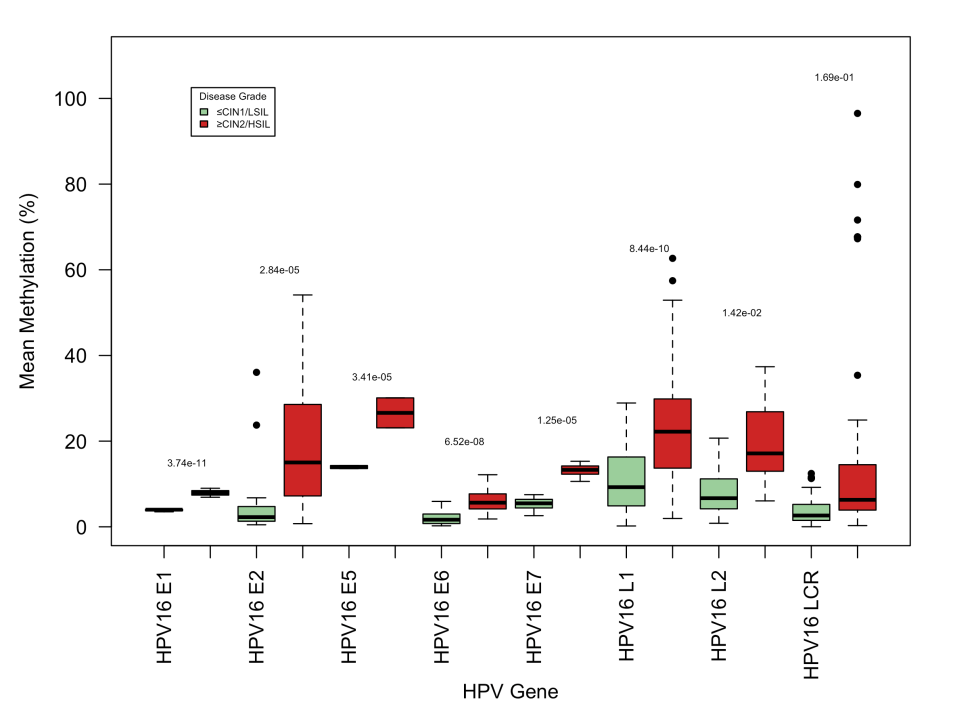
**Supplementary figure 3**. Mean methylation level in ≤CIN1/LSIL vs. ≥CIN2/HSIL for each individual study, by HPV gene. Each boxplot represents the median and interquartile range of the proportion of hyper-methylation per study, averaged by gene and grouped by disease grade. Whiskers represent 95% confidence interval bounds. Black dots represent outlier values. Black text represents P-values obtained by student’s t-test comparing mean methylation level in ≤CIN1/LSIL (green) vs ≥CIN2/HSIL (red). HPV genes ordered according to position in HPV genome but not distributed by actual gene size. ≤CIN1/LSIL: normal, ASCUS, LSIL, CIN1; ≥CIN2/HSIL: CIN2, CIN3, HSIL, ICC. a) HPV16 b) Other HPV types


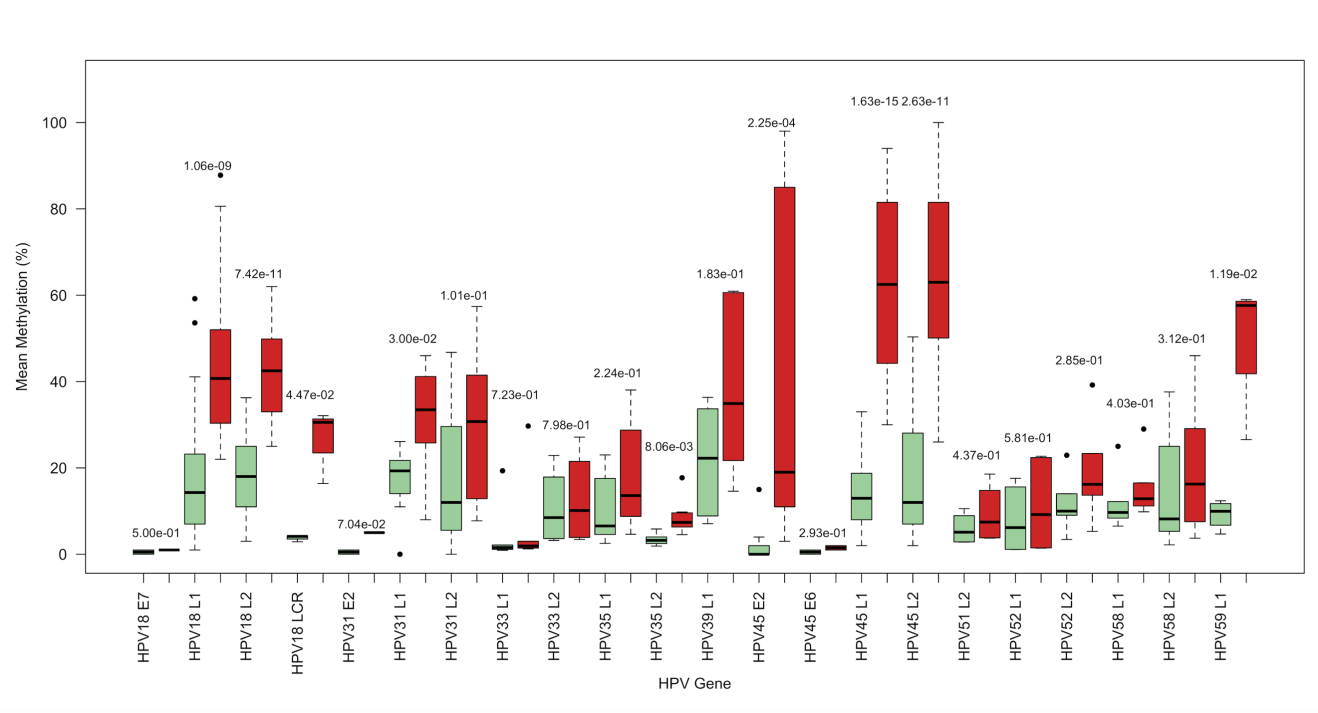


b)

a)


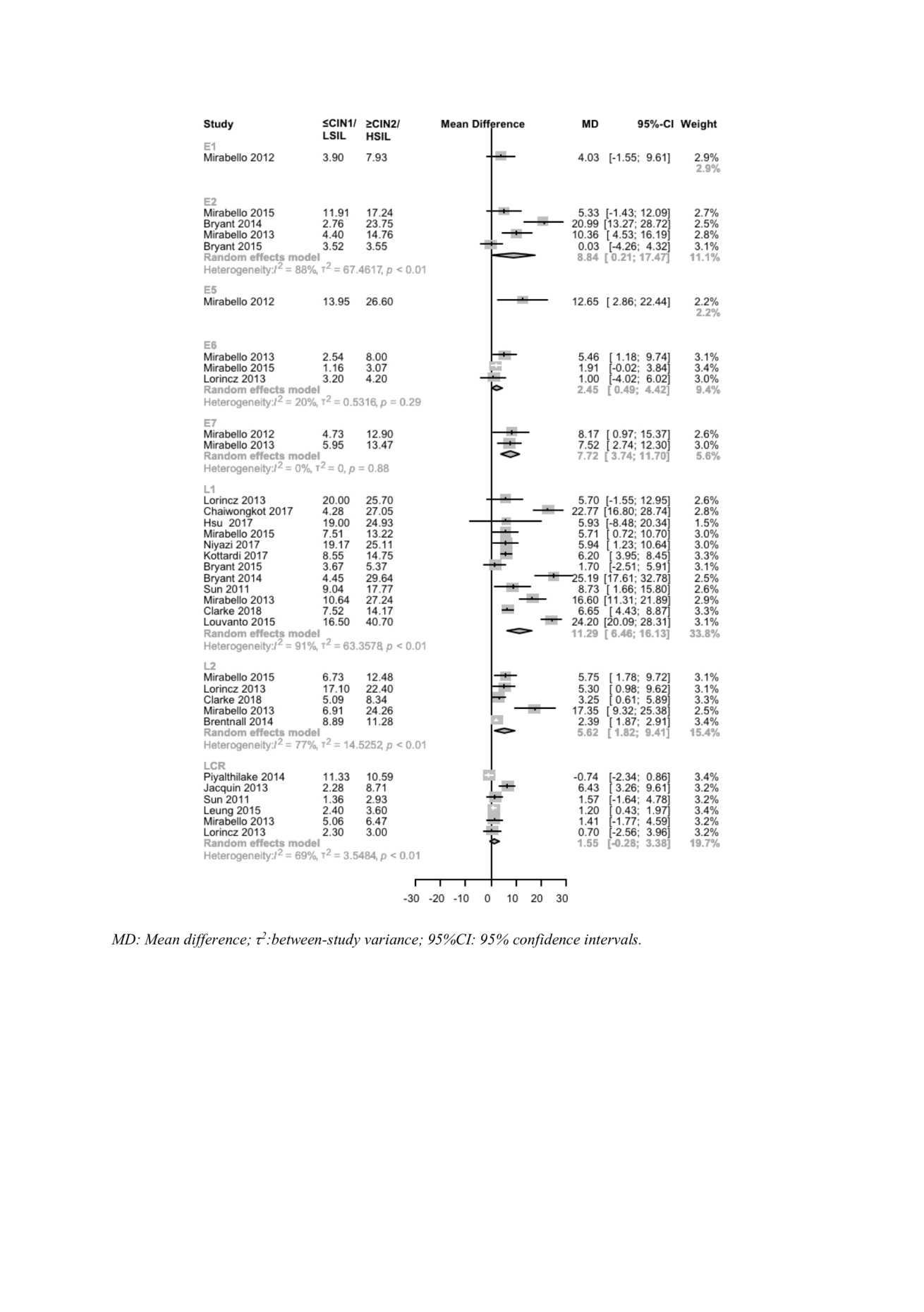
**Supplementary figure 4.** Meta-analysis of pooled difference in HPV16 mean methylation level (MD) between ≤CIN1/LSIL vs ≥CIN2/HSIL in E1, E2, E5, E6, E7, L1, L2 and LCR genes.

**Supplementary figure 5.** Subgroup meta-analysis of pooled odds ratios for HPV16 methylation in genes L1, L2, LCR, E2, E6 for ≤CIN1/LSIL vs. ≥CIN2/HSIL.

**
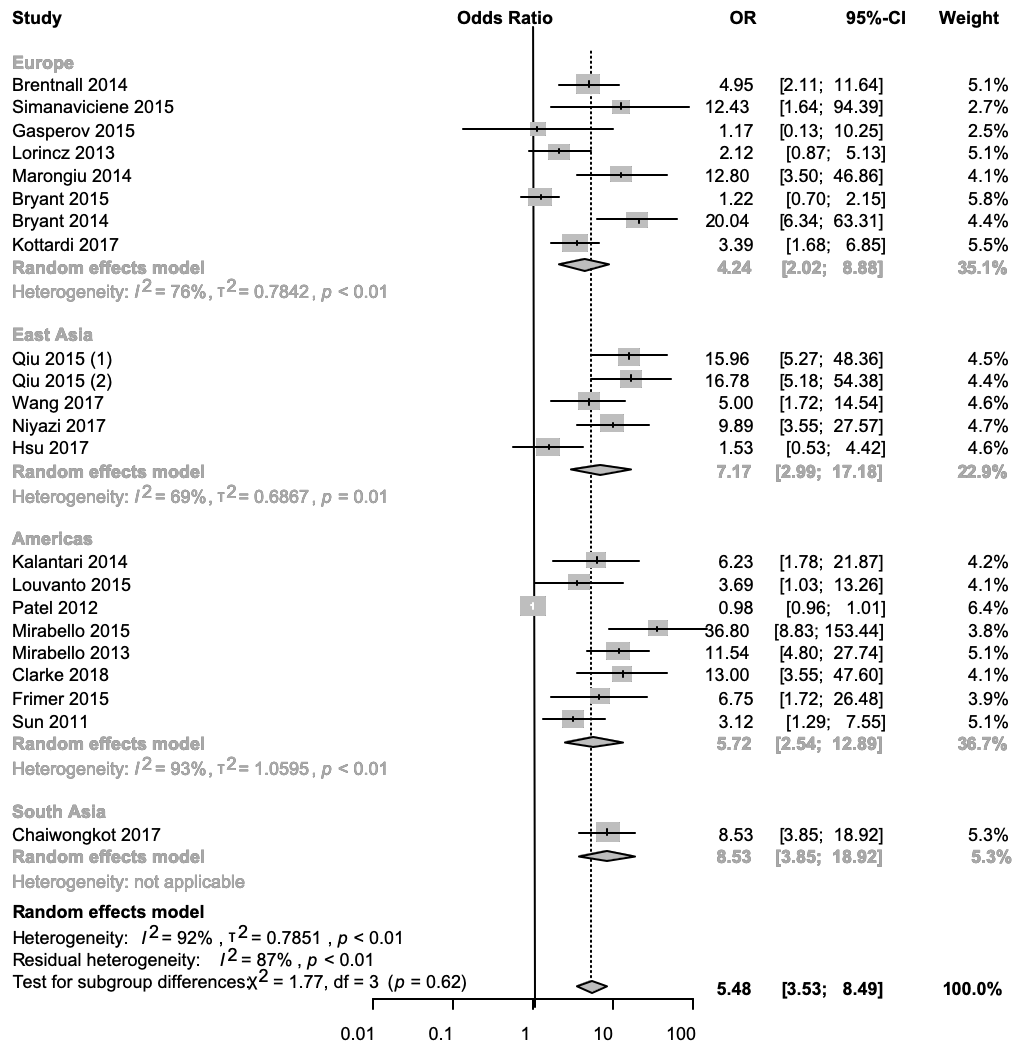
**

**Supplementary figure 6.** Diagnostic accuracy meta-analysis of HPV16 L1/L2/E2/E5/E6/E7/L1/L2/LCR in prediction of CIN2+ (univariate model): specificity, sensitivity, AUC and summary ROC curve

AUC: area under the curve; I^2^: heterogeneity; Q: Cochrane Q test; SROC: summary receiver operating curve; 95%CI: 95% confidence interval

**Supplementary figure 7.** Diagnostic accuracy meta-analysis of HPV16 L1/L2/E5 in prediction of CIN2+ (bivariate model). Sensitivity, specificity and summary ROC**.**

AUC: area under the curve; I^2^: heterogeneity; Q: Cochrane Q test; SROC: summary receiver operating curve; 95%CI: 95% confidence interval

**Supplementary figure 8.** Diagnostic Accuracy of HPV16 L1 in prediction of CIN2+ (bivariate model): Sensitivity, specificity and summary ROC.

AUC: area under the curve; I^2^: heterogeneity; Q: Cochrane Q test; SROC: summary receiver operating curve; 95%CI: 95% confidence interval

**Supplementary figure 9.** Diagnostic Accuracy of HPV16 L2 in prediction of CIN2+ (univariate model): Specificity, sensitivity and summary ROC.

AUC: area under the curve; I^2^: heterogeneity; Q: Cochrane Q test; SROC: summary receiver operating curve; 95%CI: 95% confidence interval

**Supplementary figure 10.** Diagnostic Accuracy of HPV16+ L1/L2/LCR in prediction of CIN2/HSIL+ (bivariate model): Specificity, sensitivity and summary ROC.

RE: random effects

**Supplementary figure 11.** Diagnostic Accuracy of HPV16 LCR in prediction of CIN2+ (bivariate model): Specificity, sensitivity and summary ROC.

**
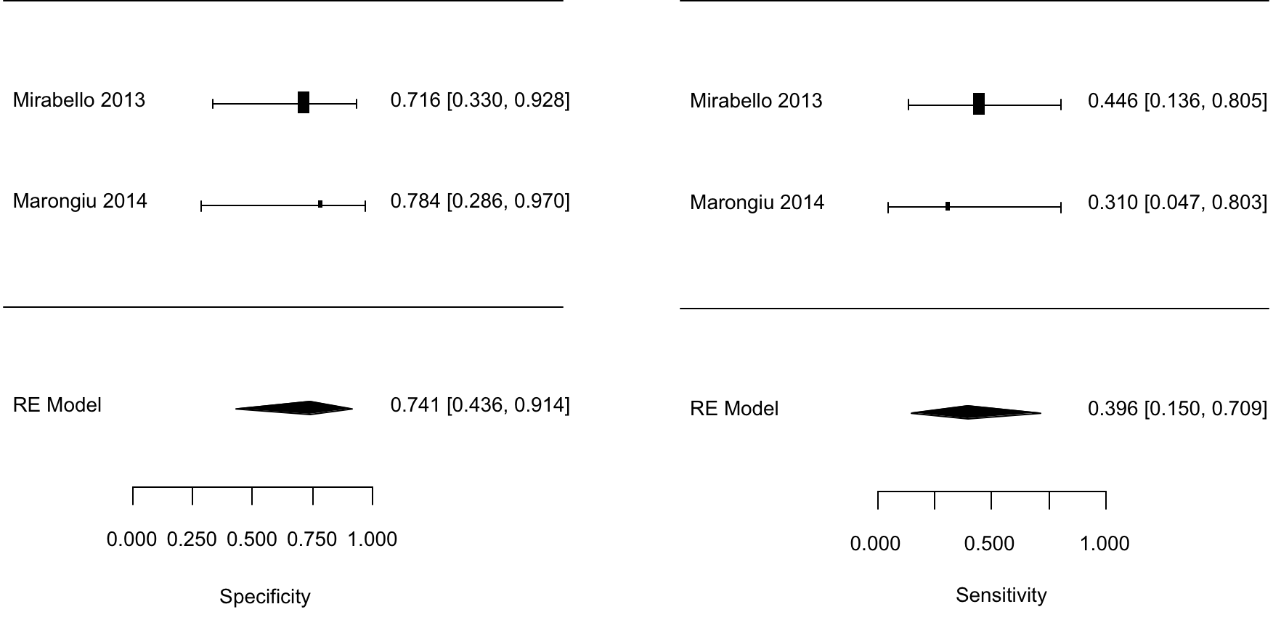

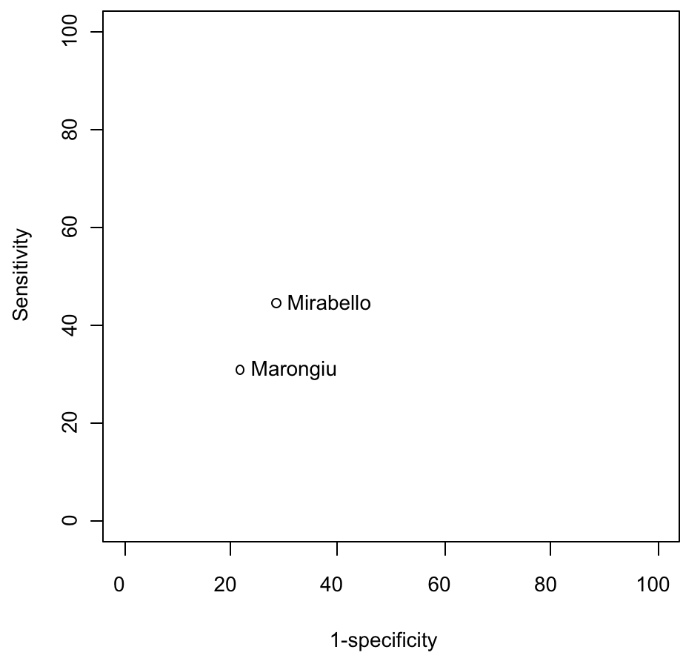
**

RE: random effects

**
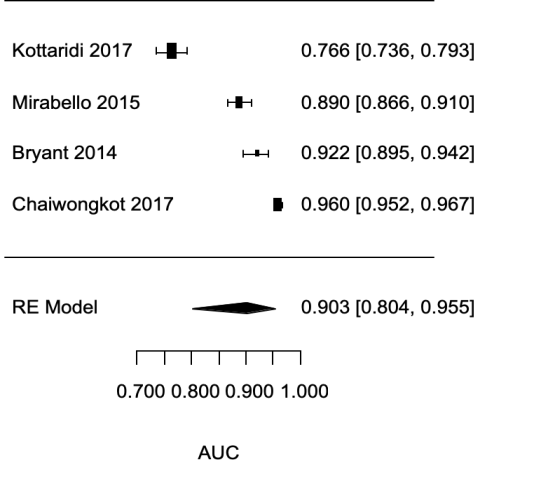

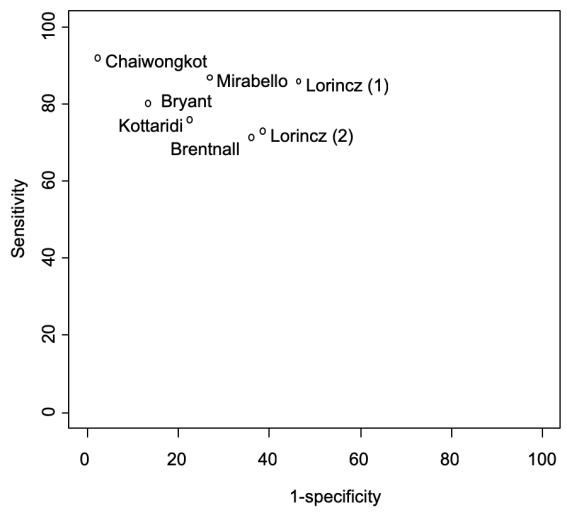
Supplementary figure 12.** Diagnostic Accuracy of HPV16 L1 in predicting CIN3+ (univariate): Sensitivity, specificity, AUC, summary ROC. (univariate model applied as Hessian matrix was not positive therefore between-study correlation of estimate -1 causing the determinant to be zero ( Riley RD, Abrams KR, Sutton AJ, Lambert PC, Thompson JR. Bivariate random-effects meta-analysis and the estimation of between-study correlation. BMC Med Res Methodol. 2007;7:3).


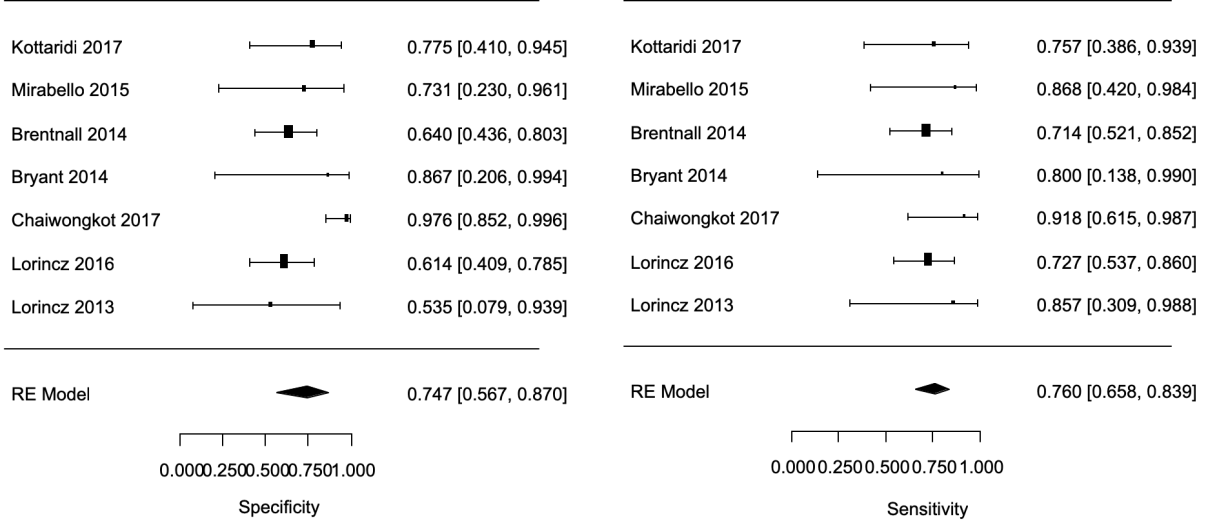
 AUC: area under the curve; RE: random effects

**Supplementary figure 13.** Diagnostic accuracy of HPV16 L1/L2 in prediction of CIN2+: Sensitivity analyses restricted to studies with low Risk of Bias (bivariate model)

AUC: area under the curve; I^2^: heterogeneity; Q: Cochrane Q test; SROC: summary receiver operating curve; 95%CI: 95% confidence interval

**Supplementary figure 14.** Diagnostic accuracy of HPV16 L1/L2 in prediction of CIN2+: Sensitivity analyses restricted to Histology only samples (univariate model applied as Hessian matrix was not positive therefore between-study correlation of estimate -1 causing the determinant to be zero)


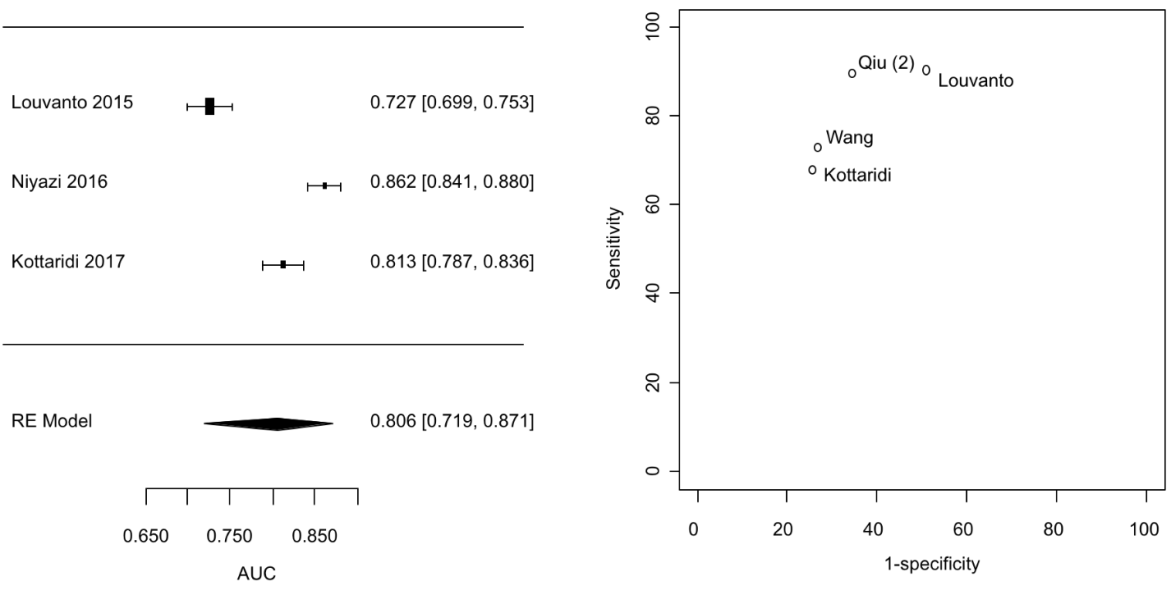
****AUC: area under the curve; RE: random effects.

**Supplementary figure 15.** HPV16 L1/L2 in prediction of CIN2+: Sensitivity analyses restricted for Pyrosequencing only samples (bivariate model)

AUC: area under the curve; I^2^: heterogeneity; Q: Cochrane Q test; SROC: summary receiver operating curve; 95%CI: 95% confidence interval

**Supplementary figure 16.** HPV16 L1/L2 in prediction of CIN2+: Sensitivity analysis restricted for exfoliated cervical cell only samples (bivariate model)

AUC: area under the curve; I^2^: heterogeneity; Q: Cochrane Q test; SROC: summary receiver operating curve; 95%CI: 95% confidence interval

**Supplementary figure 17.** HPV16 L1/L2 in prediction of CIN2+: Sensitivity analysis restricted for 10% cut-off (bivariate model)

**
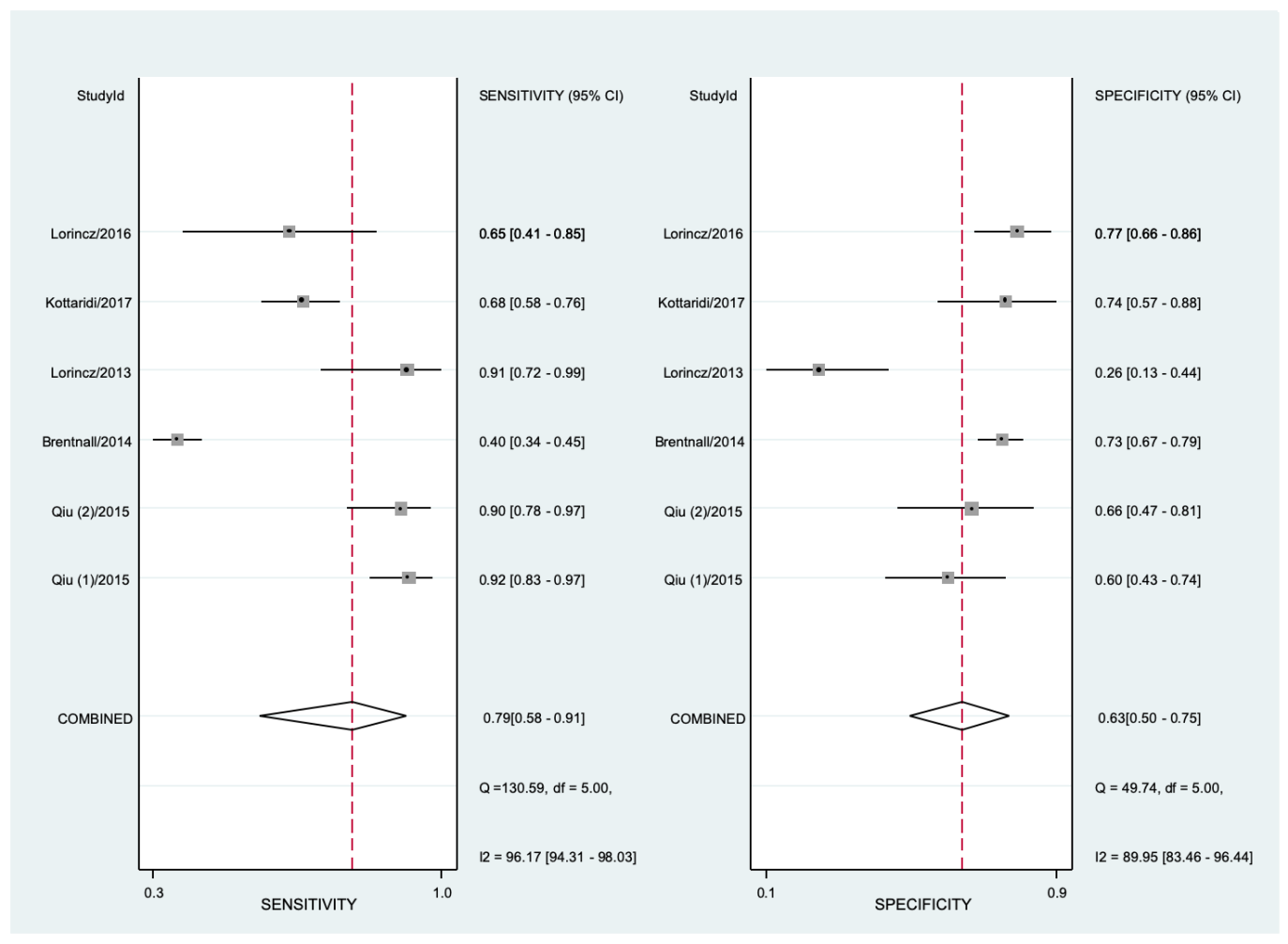
**

AUC: area under the curve; I^2^: heterogeneity; Q: Cochrane Q test; SROC: summary receiver operating curve; 95%CI: 95% confidence interval

**Supplementary figure 18:** AUC values for methylation in all HPV genotypes for prediction of a) CIN2+ and b) CIN3+

**HPV16**

**
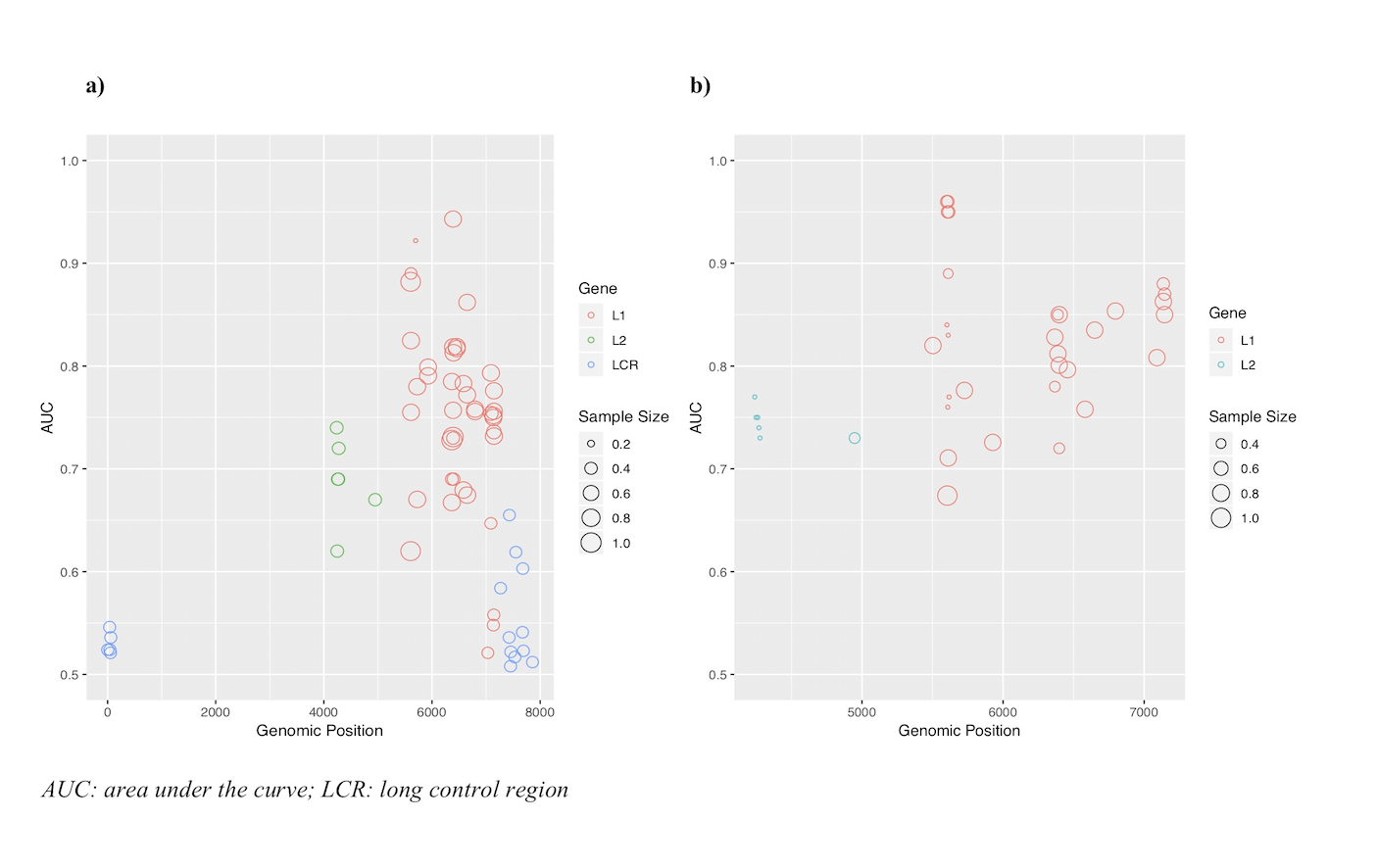
**

**HPV18**

1. b)

**HPV31**

1. b)

**HPV33**

a) b)

**HPV 45**

a) b)

**HPV 52**

1. b)

Additional References

1. Richart RM. A modified terminology for cervical intraepithelial neoplasia. Obstet Gynecol 1990; **75**(1): 131-3.

2. Solomon D, Davey D, Kurman R, et al. The 2001 Bethesda System: terminology for reporting results of cervical cytology. Jama 2002; **287**(16): 2114-9.

3. Darragh TM, Colgan TJ, Cox JT, et al. The Lower Anogenital Squamous Terminology Standardization Project for HPV-Associated Lesions: background and consensus recommendations from the College of American Pathologists and the American Society for Colposcopy and Cervical Pathology. Archives of pathology & laboratory medicine 2012; **136**(10): 1266-97.

4. Arbyn M, Verdoodt F, Snijders PJ, et al. Accuracy of human papillomavirus testing on self-collected versus clinician-collected samples: a meta-analysis. Lancet Oncology 2014; **15**(2): 1474-5488 (Electronic).

5. Whiting PF, Rutjes AW, Westwood ME, et al. QUADAS-2: a revised tool for the quality assessment of diagnostic accuracy studies. Annals of internal medicine 2011; **155**(8): 529-36.

6. Deeks JJ WS, Davenport C. Chapter 4: Guide to the contents of a Cochrane Diagnostic Test Accuracy Protocol. pp4-14. In: Deeks JJ, Bossuyt PM, Gatsonis C (editors), Cochrane Handbook for Systematic Reviews of Diagnostic Test Accuracy Version 1.0.0. The Cochrane Collaboration 2013.

7. Marongiu L, Godi A, Parry JV, Beddows S. Human Papillomavirus 16, 18, 31 and 45 viral load, integration and methylation status stratified by cervical disease stage. BMC Cancer 2014; **14**: 384.

8. Mirabello L, Schiffman M, Ghosh A, et al. Elevated methylation of HPV16 DNA is associated with the development of high grade cervical intraepithelial neoplasia. Int J Cancer 2013; **132**(6): 1412-22.

9. Kottaridi C, Kyrgiou M, Pouliakis A, et al. Quantitative Measurement of L1 Human Papillomavirus Type 16 Methylation for the Prediction of Preinvasive and Invasive Cervical Disease. J Infect Dis 2017; **215**(5): 764-71.

10. Mirabello L, Sun C, Ghosh A, et al. Methylation of human papillomavirus type 16 genome and risk of cervical precancer in a Costa Rican population. Journal of the National Cancer Institute 2012; **104**(7): 556-65.

11. Mirabello L, Frimer M, Harari A, et al. HPV16 methyl-haplotypes determined by a novel next-generation sequencing method are associated with cervical precancer. Int J Cancer 2015; **136**(4): E146-53.

12. Wan X, Wang W, Liu J, Tong T. Estimating the sample mean and standard deviation from the sample size, median, range and/or interquartile range. BMC Med Res Methodol 2014; **14**: 135.

13. Furukawa TA, Barbui C, Cipriani A, Brambilla P, Watanabe N. Imputing missing standard deviations in meta-analyses can provide accurate results. Journal of clinical epidemiology 2006; **59**(1): 7-10.

14. Rohatgi A. WebPlotDigitizer V4.1. 2018. <http://arohatgi.info/WebPlotDigitizer/>.

15. Van Doorslaer K, Li Z, Xirasagar S, et al.

16. Brentnall AR, Vasiljevic N, Scibior-Bentkowska D, et al. HPV33 DNA methylation measurement improves cervical pre-cancer risk estimation of an HPV16, HPV18, HPV31 and EPB41L3 methylation classifier. Cancer Biomarkers 2015; **15**(5): 669-75.

17. Brentnall AR, Vasiljevic N, Scibior-Bentkowska D, et al. A DNA methylation classifier of cervical precancer based on human papillomavirus and human genes. International Journal of Cancer 2014; **135**(6): 1425-32.

18. Higgins J, Green Se. Cochrane Handbook for Systematic Reviews of Interventions 5.1.0 [updated March2011: The Cochrane Collaboration; 2011.

19. Huedo-Medina TB, Sanchez-Meca J, Marin-Martinez F, Botella J. Assessing heterogeneity in meta-analysis: Q statistic or I2 index? Psychological methods 2006; **11**(2): 193-206.

20. Cochrane W. The combination of estimates from different experiments. Biometrics 1954; **10**: 101-29.

21. Barendregt JJ, Doi SA, Lee YY, Norman RE, Vos T. Meta-analysis of prevalence. J Epidemiol Community Health 2013; **67**(11): 974-8.

22. Freeman M, Tukey J. Transformations Related to the Angular and the Square Root. Ann Math Statist 1950; **21**(4): 607-11.

23. Miller JJ. The Inverse of the Freeman – Tukey Double Arcsine Transformation. The American Statistician 1978; **32**(4): 138.

24. Borenstein M, Hedges LV, Higgins JPT, Rothstein HR. Introduction to Meta-analysis: Wiley; 2009.

25. Dwamena B. MIDAS: Stata Module for Meta-analytical Integration of Diagnostic Test Accuracy Studies. . MIDAS: Stata module for meta-analytical integration of diagnostic test accuracy studies 2009.

26. Viechtbauer W. Conducting meta-analyses in R with the metafor package. Journal of Statistical Software 2010; **36**(3): 1-48.

27. DerSimonian R, Laird N. Meta-analysis in clinical trials. Controlled clinical trials 1986; **7**(3): 177-88.

28. Raudenbush S. Analyzing effect sizes: Random-effects models. In: H C, LV H, JC V, eds. The handbook of research synthesis and meta-analysis

. New York: The Russel Sage Foundation; 2009: 295–316.

29. Langan D, Higgins JPT, Jackson D, et al. A comparison of heterogeneity variance estimators in simulated random‐effects meta‐analyses. Res Syn Meth 2018: 1-16.

30. Viechtbauer W. Confidence intervals for the amount of heterogeneity in meta-analysis. Stat Med 2007; **26**(1): 37-52.

31. Arbyn M, Xu L, Verdoodt F, et al. Genotyping for Human Papillomavirus Types 16 and 18 in Women With Minor Cervical Lesions: A Systematic Review and Meta-analysis. Annals of internal medicine 2017; **166**(2): 118-27.

32. R_Core_Team. R: A language and environment for statistical computing. . Vienna, Austria: R Foundation for Statistical Computing; 2019.

33. StataCorp. Stata Statistical Software: Release 14. College Station, Texas, USA: StataCorp LP; 2015.

34. Lever. HPV Methylation for Diagnosis of Cervical Intraepithelial Neoplasia. 2018. <https://osf.io/c6k29/>.
